# Herbst and Twin Block appliances in Class II malocclusion management for children: a systematic review and meta-analysis

**DOI:** 10.3389/fdmed.2026.1717387

**Published:** 2026-05-15

**Authors:** Franz Tito Coronel-Zubiate, Joan Manuel Meza-Málaga, Sara Antonieta Luján-Valencia, Consuelo Marroquín-Soto, Fredy Cruzado-Oliva, Rubén Aguirre-Ipenza, Eduardo Luján-Urviola, Carlos Alberto Farje-Gallardo, Ary dos Santos Pinto, Heber Isac Arbildo-Vega

**Affiliations:** 1 Faculty of Health Sciences, Stomatology School, Universidad Nacional Toribio Rodríguez de Mendoza de Amazonas, Chachapoyas, Peru; 2 Faculty of Dentistry, Dentistry School, Universidad Católica de Santa María, Arequipa, Peru; 3 Faculty of Dentistry of Araraquara, Dentistry Science Graduate Program-Orthodontics, São Paulo State University “Júlio de Mesquita Filho” (UNESP), Araraquara, Sao Paulo, Brazil; 4 Department of Diagnostics and Surgery, School of Dentistry, Paulista State University Julio de Mesquita Filho (UNESP), Araraquara, Sao Paulo, Brazil; 5 Department of Dentistry, School of Dentistry, Universidad Científica del Sur, Lima, Peru; 6 Faculty of Stomatology, Stomatology School, Universidad Nacional de Trujillo, Trujillo, Peru; 7 Faculty of Health Sciences, Universidad Continental, Lima, Peru; 8 Faculty of Dentistry, Universidad Andina Néstor Cáceres Velásquez, Juliaca, Peru; 9 Faculty of Dentistry of Araraquara, Department of Morphology and Pediatric Clinic—Orthodontics, São Paulo State University “Júlio de Mesquita Filho” (UNESP), Araraquara, Sao Paulo, Brazil; 10 Faculty of Dentistry, Dentistry School, Universidad San Martín de Porres, Chiclayo, Peru; 11 Faculty of Human Medicine, Human Medicine School, Universidad San Martín de Porres, Chiclayo, Peru; 12 Posgraduate School, Universidad Nacional Toribio Rodríguez de Mendoza de Amazonas, Chachapoyas, Peru

**Keywords:** Class II malocclusion, functional orthodontic appliances, growing patients, Herbst appliance, orthopedic treatment, Twin Block appliance

## Abstract

**Introduction:**

Class II malocclusion is a common skeletal abnormality in growing patients, often associated with mandibular retrusion. Functional appliances such as Herbst and Twin Block appliances aim to stimulate mandibular growth during puberty. However, evidence comparing their effectiveness remains inconsistent.

**Objective:**

To compare the skeletal, dental, and soft tissue effects of Herbst and Twin Block appliances in growing patients with Class II malocclusion through a systematic review and meta-analysis.

**Methods:**

Randomized controlled trials (RCTs) comparing both appliances in children ≤16 years of age with Class II malocclusion were included if they reported skeletal, dental, or soft tissue outcomes. Non-randomized studies, case reports, reviews, and those with incomplete data were excluded. PubMed, Scopus, Web of Science, the Cochrane Library, and EMBASE, among other sources, were searched until January 30, 2025, with no language or date restrictions. Risk of bias was assessed using the Cochrane RoB 2.0 tool, and the certainty of the evidence was evaluated using the GRADE method. A random-effects meta-analysis calculated standardized mean differences (SMDs) with 95% confidence intervals.

**Results:**

Seven RCTs with 548 participants were included. Both appliances effectively managed Class II malocclusion, with generally similar effects on skeletal and soft tissue parameters. The Herbst appliance showed significant advantages in improving the mentolabial angle (SMD: 0.56; 95% CI: 0.15–0.96) and molar relationship (SMD: −0.31; 95% CI: −0.56 to −0.05). No significant differences were observed in other outcomes.

**Conclusions:**

Both the Herbst and Twin Block appliances are effective for Class II correction in growing patients. The Herbst appliance offers modest advantages in certain parameters, but clinical decisions should also consider patient-specific factors such as adherence, comfort, esthetics, and growth stage.

**Systematic Review Registration:**

https://www.crd.york.ac.uk/PROSPERO/view/CRD420251011083, PROSPERO: CRD420251011083.

## 1 Introduction

Class II malocclusion is one of the most prevalent orthodontic conditions in children and adolescents. It is characterized by a discrepancy in maxillomandibular growth, often due to mandibular retrusion, which leads to excessive facial convexity and compromised esthetics and function (1, 2). Treatment is most effective during the pubertal growth spurt, targeting skeletal structures through either restraint of maxillary growth or stimulation of mandibular advancement, depending on the specific clinical scenario (3, 4). In cases of significant crowding or dental arch discrepancies, adjunctive procedures such as arch expansion or selective tooth extractions may be indicated to improve alignment and occlusion (5–7).

The therapeutic approach depends on the severity of the malocclusion, the patient's skeletal maturity, and their compliance with treatment (8). Functional orthopedic appliances are essentials tools for modifying jaw growth and improving facial balance (9). Among these, the Twin Block appliance is notable for its removable design, allowing for progressive mandibular advancement in a non-invasive manner and facilitating patient adaptation (10). However, its success largely depends on consistent wear and patient cooperation, along with regular follow-up (11).

In contrast, the Herbst appliance is a fixed device that holds the mandible in a forward position, enabling continuous orthopedic stimulation and promoting mandibular remodeling (12). Its major advantage is independence from patient compliance, which can improve treatment effectiveness and reduce the need for surgical alternatives in some cases (13).

A recent meta-analysis by Xu et al. (14) compared the effects of these two appliances in children with Class II malocclusion. However, important methodological differences exist between that study and the present review, particularly regarding the use of structured evidence assessment frameworks such as the GRADE approach.

In this context, the present systematic review and meta-analysis aim to provide an updated and more comprehensive synthesis of the evidence, focusing exclusively on randomized controlled trials (RCTs) comparing Herbst and Twin Block appliances. This approach ensures high methodological rigor, enables a robust comparison of skeletal, dental, and soft tissue outcomes, and incorporates advanced assessments such as GRADE evidence profiles and sensitivity analyses. The selection of these two appliances was based on their clinical relevance and the availability of high-quality comparative data, offering a solid basis for evidence-informed clinical decision-making in growing patients with Class II malocclusion.

## 2 Materials and methods

### 2.1 Protocol and registration

This systematic review and meta-analysis were conducted in accordance with the Preferred Reporting Items for Systematic Reviews and Meta-Analyses Protocols (PRISMA-P) guidelines. The review protocol was prospectively registered in the International Prospective Register of Systematic Reviews (PROSPERO) under the registration number CRD420251011083.

### 2.2 Focused question (PICO)

The research question was formulated according to the PICO framework as follows:

- Population (P): Growing patients (≤16 years) diagnosed with Class II malocclusion and undergoing treatment with functional orthodontic appliances.
- Intervention (I): Orthodontic treatment with the Herbst appliance.
- Comparison (C): Orthodontic treatment with the Twin Block appliance.
- Outcomes (O): Changes in craniofacial morphology, dental relationships, soft tissue profile, occlusal relationships, and skeletal discrepancies.

Review question: Are Herbst appliances more efficacious than Twin Block appliances in improving skeletal, dental and soft tissue outcomes in growing patients with Class II malocclusion?

For this review, the predefined primary outcome was the sagittal correction of Class II malocclusion, specifically changes in molar relationship and mandibular advancement. Skeletal and soft tissue outcomes were designated as secondary outcomes.

### 2.3 Search strategy

A comprehensive electronic search was conducted five databases: PubMed, Cochrane Central Register of Controlled Trials (CENTRAL), EMBASE, Scopus, and Web of Science (WoS). The search strategy combined Medical Subject Headings (MeSH) and free-text terms such as: “malocclusion”, “Class II malocclusion”, “Angle Class II”, “Herbst”, “Herbst appliance”, “Twin Block”, and “Twin Block appliance”, using Boolean operators “AND” and “OR”.

To minimize publications bias, grey literature was also searched through OpenGrey and Google Scholar (first 100 results). Additionally, manual searches were performed in relevant orthodontic journals and the references list of included articles and related systematic reviews.

Duplicate records were automatically removed using Zotero. No restrictions were applied regarding language or publication date. The final search was completed on January 30, 2025. Only randomized clinical trials (RCTs) were considered eligible. The complete strategies, including Boolean operators and filters, are reported in Supplementary Table S1.

### 2.4 Eligibility criteria

Inclusion criteria:

- Study design: Randomized controlled trials (RCTs).
- Population: Children or adolescents aged ≤16 years with Class II malocclusion during the growth phase. This age threshold was selected to ensure that participants were within the period of active skeletal growth or late skeletal maturation, during which orthopedic effects of functional appliances are still achievable (15).
- Intervention and Comparison: Randomized controlled trials comparing orthodontic treatment with Herbst functional appliance (intervention) vs. Twin Block appliance (comparator) in growing patients.
- Outcomes: Studies reporting quantitative changes in craniofacial skeletal structures, dental relationships, facial angles, or soft tissue characteristics.

Exclusion criteria:

- Non-randomized studies, observational studies, case reports, reviews, editorials, or letters to the editor.
- Studies with incomplete outcome data or insufficient reporting for analysis.

### 2.5 Study selection and data extraction

Study selection was conducted independently by two reviewers (F.T.C.-Z. and F.H.C.-O.) in a two-step process: initial screening of titles and abstracts, followed by full-text evaluation of potentially eligible articles. Disagreements were resolved through discussion, and if necessary, by consultation with a third reviewer (J.M.M.-M.). The Rayyan QCRI web-based platform was used to facilitate the screening process.

To avoid double-counting of data from multiple reports of the same trial, the review team cross-referenced author names, sample sizes, trial identifiers, and methodology. When multiple publications originated from the same cohort, only the most comprehensive or relevant report was included in the meta-analysis, and others were considered supplementary sources for contextual information.

Data extraction was performed independently by two other reviewers (C.M.-S. and S.A.L.-V.) using a standardized and pre-piloted Excel spreadsheet. Extracted information included: first author, year of publication, country of study, study design, sample size, participant demographics (age and sex), type of functional appliance used (Herbst or Twin Block), duration of follow-up, and reported outcomes.

Only studies providing cephalometric assessments of skeletal and dental changes before and after treatment were included. Specific variables extracted included skeletal parameters [SNA, SNB, and ANB angles; effective mandibular length (Co-Gn); skeletal base positions], dental relationships (molar relationship, overjet, and overbite when available), and soft tissue characteristics (e.g., facial angles, lip thickness, and profile measurements). Statistical outcomes such as means and standard deviations were also recorded.

All extracted data were organized and cross-checked for consistency using Microsoft Excel. Discrepancies were resolved through consensus or, when required, arbitration by a third reviewer (H.I.A.-V.).

### 2.6 Collected variables

The review extracted methodological and clinical characteristics from each included study, including: first author, year of publication, country of origin, study design, sample size, mean participant age, and type of functional appliance used. Only studies reporting cephalometric assessments of skeletal and dental structures before and after treatment were included.

#### 2.6.1 Skeletal and dental variables

Key skeletal parameters extracted were the SNA, SNB, and ANB angles, effective mandibular length (Co-Gn), and skeletal base positions [point A and pogonion in relation to the occlusal line perpendicular (OLp)]. Composite mandibular length (pg/OLp + co/OLp) and skeletal discrepancy (A/OLp—Pg/OLp) were also recorded. Dental and occlusal variables included molar relationship measurements (is/OLp—li/OLp and ms/OLp—mi/OLp) and, when reported, changes in overjet and overbite.

#### 2.6.2 Soft tissue profile measurements

Soft tissue outcomes included angular variables such as the mentolabial angle (li–sl–pog), H-angle (Holdaway), nasolabial angle (c–sn–ls), and soft tissue convexity (na–prn–pog and na–sn–pog). Linear variables included the position of soft tissue points relative to the vertical reference line (VRL) and E-line, such as VRL–pog, VRL–li, VRL–si, VRL–ls, E–li, and E–ls. Additional soft tissue measures included lower lip length (lls–me), upper lip length (sn–uls), lip thickness, and interlabial gap.

#### 2.6.3 Outcome prioritization

The primary outcomes were defined as skeletal and dental changes relevant to the sagittal correction of Class II malocclusion, with emphasis on mandibular advancement (e.g., Co-Gn) and molar relationship. Soft tissue parameters were included as secondary outcomes, and post-treatment stability was also considered where reported.

### 2.7 Risk of bias assessment

The risk of bias of the included randomized controlled trials was independently assessed by two reviewers (HIA-V and RA-I) using the Cochrane Risk of Bias 2.0 (RoB 2.0) tool. Disagreements were resolved through discussion and consensus. A visual summary of the domain-specific and overall risk assessments was generated (see Figure 2).

### 2.8 Data synthesis and analysis

A qualitative synthesis was carried out to describe the methodological characteristics of the included studies. Where sufficient data were available, a meta-analysis was conducted using RevMan 5.3 (Cochrane Collaboration), applying a random-effects model. Between-study variance was estimated using the Paule–Mandel method. Effect sizes were planned to be reported as standardized mean differences (SMDs) with 95% confidence intervals (CIs).

Statistical heterogeneity was intended to be assessed using Cochran's *Q*-test and the *I*^2^ statistic, with *I*^2^ values greater than 50% considered indicative of substantial heterogeneity. Pre-specified subgroup analyzes were planned based on appliance type, follow-up duration, or patient age, depending on data availability.

Potential publication bias was to be explored through funnel plot inspection and Egger's regression test. The certainty of the evidence for each outcome was evaluated using the GRADE approach, considering five domains: risk of bias, inconsistency, indirectness, imprecision, and publication bias. Judgments were made independently by two reviewers and documented in GRADEpro GDT, with reasons for downgrading explicitly stated.

## 3 Results

### 3.1 Selection of studies

**Figure 1:**
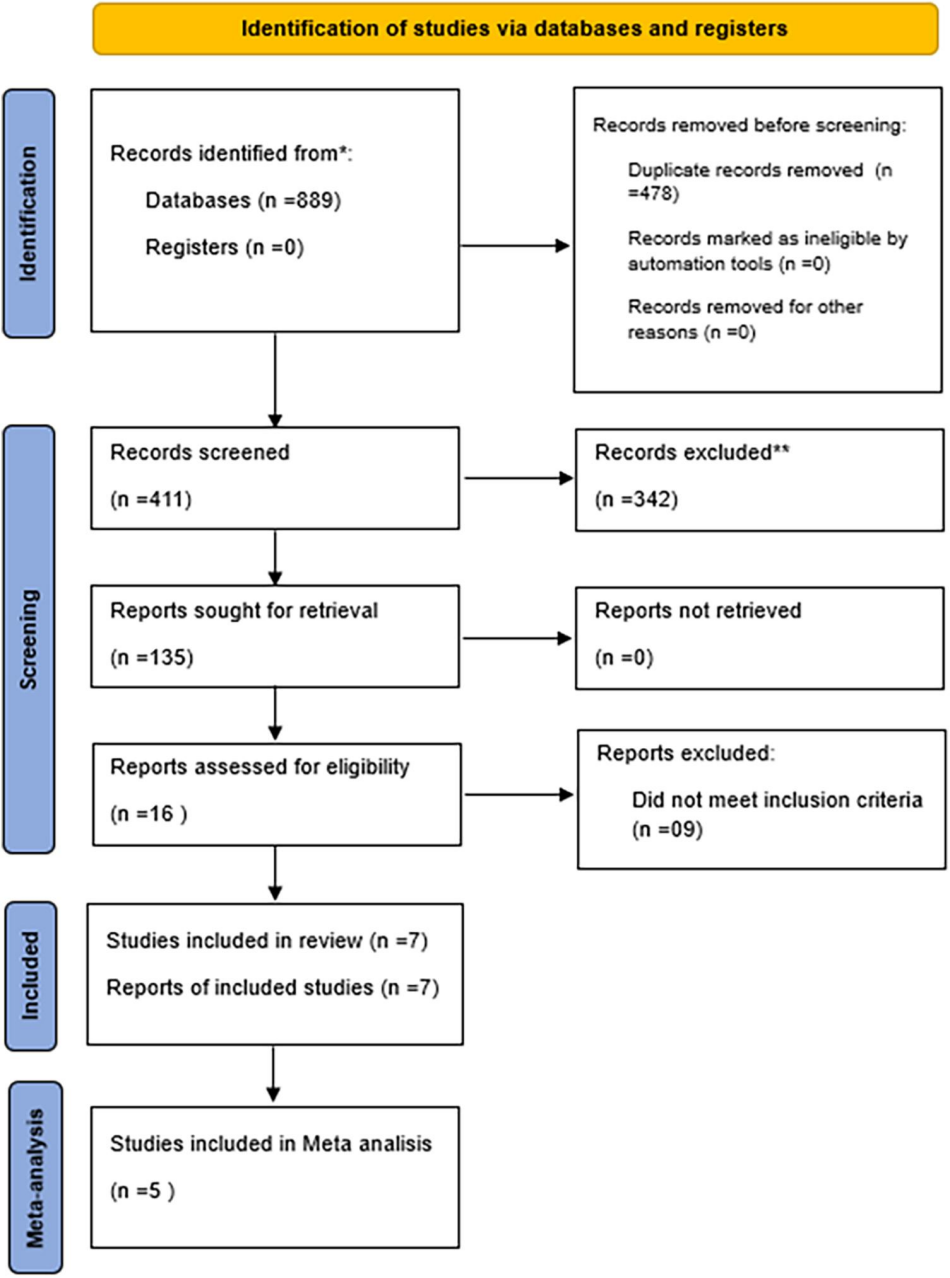
PRISMA 2020 flow diagram of the study selection process.

The selection process and descriptive characteristics of the included studies are summarized below. The initial search yielded 889 records. After removing 478 duplicates using Zotero, 411 records were screened based on titles and abstracts. From these, 135 reports were sought for full-text retrieval, of which 16 were assessed for eligibility. Finally, 7 studies met the inclusion criteria and were included in the qualitative synthesis, and 5 of these were eligible for inclusion in the meta-analysis. The study selection process is detailed in Figure 1 (PRISMA flowchart). Multiple reports from the same clinical trial were identified during screening and treated as a single unit of analysis to prevent data duplication.

### 3.2 Study characteristics

Skeletal age was explicitly reported in three studies (16–18), ranging from 11.45 to 12.26 years. Participants in the included studies were in the growth phase, with baseline chronological ages ranging from approximately 11.8 to 13.1 years (18, 19). All studies included both male and female participants.

Follow-up durations ranged from 3 to 16.2 months, depending on the appliance used and the study protocol (16–22). All trials reported outcomes assessed at baseline (T0) and post-treatment (T1), employing cephalometric analysis, cone-beam computed tomography (CBCT), or clinical evaluations, depending on the study.

Key characteristics of individual studies include:

- Baysal & Uysal (16, 17) Included an untreated control group and evaluated outcomes over 15–16 months.
- Brandão et al. (18) assessed skeletal changes using cone-beam computed tomography (CBCT) after a 12-month follow-up, comparing two Herbst appliance designs: Herbst with double anchorage (HDA) and Herbst with skeletal anchorage (HSA).
- Güler & Malkoc (19): Included a monoblock appliance group and followed participants for 9 months.
- O'Brien et al. (20): Had the largest total sample (*n* = 215), with participants randomized to Herbst, Twin Block, or control groups, and reported detailed demographic data.
- Pacha et al. (21, 22): Conducted two sequential trials comparing Hanks Herbst and Twin Block appliances, with follow-ups of 3 and 12 months, respectively.

The detailed characteristics of the included randomized controlled trials are presented in Supplementary Table S3.

You may insert up to 5 heading levels into your manuscript as can be seen in “Styles” tab of this template. These formatting styles are meant as a guide, as long as the heading levels are clear, Frontiers style will be applied during typesetting.

### 3.3 Risk of bias analysis of studies

Seven randomized controlled trials were evaluated: Baysal & Uysal (16), Baysal & Uysal (17), Brandão et al. (18), Güler & Malkoc (19), O'Brien et al. (20), Pacha et al. (21), and Pacha et al. (22).

Five of these studies were judged to have a low overall risk of bias, while two studies Baysal & Uysal (16, 17) were rated as presenting “some concerns”, primarily due to potential deviations from intended interventions and limitations in outcome measurement protocols.

No study was assessed as having a high risk of bias.

**Figure 2:**
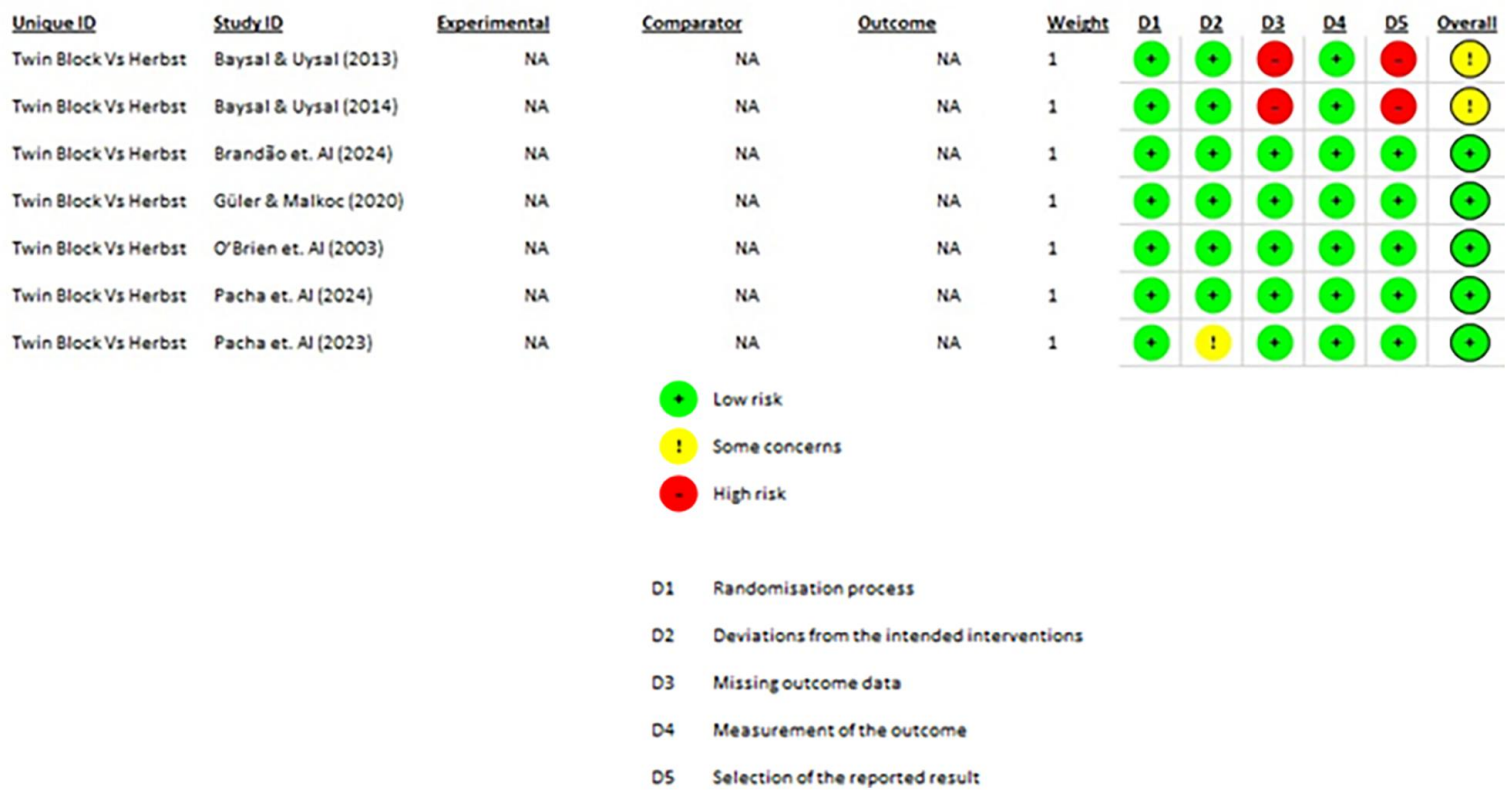
Risk of bias.

This variability was taken into account when interpreting the findings and was reflected in the GRADE assessment of the certainty of evidence. A summary of the risk of bias assessment is presented in Figure 2.

### 3.4 Sensitivity analysis

To evaluate the robustness of the meta-analytic findings, a sensitivity analysis was conducted by applying a fixed-effects model and comparing the resulting outcomes to those obtained with the random-effects model, which was used as the primary analytical approach throughout this review.

Some variables—specifically soft tissue convexity (na-prn-pog), vertical reference line to subnasale (VRL–si), and vertical reference line to pogonion (VRL–pog)—exhibited statistically significant differences under the fixed-effects model that were not maintained when analyzed using the random-effects model.

This discrepancy suggests that these particular outcomes may be sensitive to the statistical model applied and potentially influenced by heterogeneity across studies. Although the effect sizes observed were moderate, their clinical relevance should be interpreted with caution, given the lack of statistical consistency.

A detailed summary of the sensitivity analysis comparing fixed-effects and random-effects models for selected variables is provided in Supplementary Table S4.

### 3.5 Synthesis of results

To aid clinical interpretation of the pooled effect sizes, reference values for each cephalometric and soft tissue variable are provided in Supplementary Table S5. Normal Reference Values for Skeletal and Soft Tissue Parameters. These normative values, derived from the included studies and standard cephalometric ranges, serve as a benchmark to evaluate the magnitude and potential clinical relevance of observed differences.

#### 3.5.1 Skeletal and craniofacial changes

A meta-analysis of 2 to 3 studies per variable revealed no statistically significant differences between the Herbst and Twin Block appliances across most evaluated skeletal parameters. Specifically, no significant differences were found in composite mandibular length (pg/OLp + Co/OLp) (SMD = −0.07, 95% CI: −0.47 to 0.33; *p* = 0.73; *I*^2^ = 58%) or in pogonion position (SMD = −0.34, 95% CI: −0.86 to 0.17; *p* = 0.19; *I*^2^ = 58%).

Similarly, the meta-analysis showed no statistically significant differences in the position of the maxillary base (A/OLp) or mandibular base (pg/OLp). These findings indicate that both appliances produce comparable anteroposterior skeletal changes in growing patients with Class II malocclusion.

#### 3.5.2 Dental and occlusal outcomes

A statistically significant difference favoring the Herbst appliance was found for the sagittal molar relationship, measured as the molar relationship (is/OLp—Li/OLp) (SMD = −0.31, 95% CI: −0.56 to −0.05; *p* = 0.02; *I*^2^ = 6%), indicating superior correction of molar relationship in patients treated with Herbst. Supplementary Table S6 presents the baseline cephalometric dental measurements reported in the included studies.

Conversely, no statistically significant differences were observed in other dental parameters, such as the lower incisor position (si–B) or skeletal discrepancy (A/OLp—Pg/OLp). These findings suggest that, although both appliances achieve comparable outcomes in general occlusal parameters, the Herbst appliance may exert a more pronounced effect on molar relationship correction. A comprehensive summary of all meta-analysis outcomes, including soft tissue, skeletal, and dental variables, is represented in Supplementary Table S7.

#### 3.5.3 Soft tissue profile and facial changes

Changes in the soft tissue profile were evaluated in multiple studies. A statistically significant difference favoring the Herbst appliance was observed in the mentolabial angle (li–sl–pog) (SMD = 0.56, 95% CI: 0.15 to 0.96; *p* = 0.007; *I*^2^ = 0%), indicating a potentially more favorable aesthetic effect in the lower third of the face.

However, no significant differences were found between appliances in other soft tissue parameters, including soft tissue convexity (na–prn–pog), H angle, nasolabial angle, lip length, and lip thickness. Further details on the specific maxillary and mandibular soft tissue measurements assessed in the included studies are provided in Supplementary Tables S8, S9. These findings suggest that while both appliances have comparable effects on overall soft tissue outcomes, the Herbst appliance may confer a specific aesthetic advantage in the mentolabial region. A comprehensive summary of all meta-analysis outcomes, including soft tissue, skeletal, and dental variables, is represented in Supplementary Table S7.

A visual representation of the meta-analytical results for all evaluated outcomes is provided in Supplementary Figure S1 (Forest Plots of All Outcomes).

### 3.6 GRADE analysis

The certainty of evidence was assessed for each outcome using the GRADE approach. The results showed high certainty for the mentolabial angle and molar relationship outcomes, indicating robust and consistent findings across studies. A full GRADE evidence profile for all evaluated outcomes is available in Supplementary Table S10, and a narrative summary of selected key findings is provided in Supplementary Table S11. Specifically:

- The mentolabial angle (li–sl–pog) was supported by three RCTs with no serious limitations across domains, resulting in high certainty.
- The molar relationship (is/OLp—li/OLp) also demonstrated high certainty, with consistent results in two well-conducted trials.
- In contrast, moderate to very low certainty was assigned to other outcomes due to issues such as imprecision and inconsistency:
- The H angle presented moderate certainty due to some concerns regarding imprecision.
- Outcomes such as upper lip thickness and condylar head position (co/OLp) were rated with very low certainty, primarily due to serious concerns in both inconsistency and imprecision.

These findings suggest that while some skeletal and soft tissue effects of the appliances are supported by strong evidence, others require further well-designed studies to improve the reliability of the conclusions.

Reasons for downgrading included serious imprecision (wide confidence intervals, small sample sizes), and inconsistency (high heterogeneity or conflicting results), as detailed in Supplementary Table S10.

## 4 Discussion

In this systematic review with meta-analysis, multiple cephalometric and soft tissue parameters were analyzed to compare the effects of Herbst and Twin Block functional appliances in the treatment of Class II malocclusion. Variables evaluated included facial convexity (na-prn-pog, na-sn-pog), H-angle, nasolabial angle (c-sn-ls), mentolabial angle (li-sl-pog), various linear soft tissue measurements (e.g., VRL-prn, VRL-sn, VRL-ss, VRL-ls, E-ls, VRL-li, VRL-si, E-li, VRL-pog, Pog-pog), labial thickness and length, interlabial space, as well as dental and skeletal parameters such as molar relationship (is/OLp—li/OLp and ms/OLp—mi/OLp), maxillary and mandibular base position, skeletal discrepancy, condylar head position, and composite mandibular length. Although several evaluated outcomes did not show statistically significant differences between appliances under the random-effects model, the overall results suggest that both mechanisms induce comparable changes in craniofacial morphology. Clinically, this implies that neither appliance demonstrates superior overall efficacy under controlled conditions, supporting an individualized approach to appliance selection.

Sensitivity analysis revealed that soft tissue convexity (na-prn-pog), VRL-si, and VRL-pog were statistically significant in favour of Twin Block under fixed-effects, but lost significance under random-effects, reflecting the influence of heterogeneity and limiting the robustness of these findings. The effect size of soft tissue convexity (SMD ≈ −0.44) is small and unlikely to be visually perceptible. Similarly, the displacement in VRL-si (SMD ≈ −0.52) and VRL-pog (SMD ≈ −0.54) may not be clinically relevant or noticeable, reducing their practical importance. These nuances suggest that although some outcomes slightly favor Twin Block under restrictive statistical assumptions, the magnitude of these differences is insufficient to warrant appliance preference in clinical settings.

Among soft tissue variables, the mentolabial angle (li-sl-pog) favoured Herbst [SMD: 0.56 (0.15, 0.96); *p* = 0.007], aligning with findings from Xu et al. (14), who reported enhanced mandibular growth with Herbst. Similarly, the molar relationship (is/OLp—li/OLp) significantly improved with Herbst [SMD: −0.31 (−0.56, −0.05); *p* = 0.02], suggesting more effective sagittal correction. However, no significant differences were found in nasolabial angles, general convexity, or vertical parameters. Xu et al. (14) found greater aesthetic improvement with Twin Block, which contrasts with our results and may be due to differences in assessment techniques or sample characteristics.

However, these discrepancies may also be explained by methodological differences between the present review and that of Xu et al. (14). In their meta-analysis, the inclusion criteria encompassed both randomized and non-randomized studies, which may have increased the risk of bias and affected the internal validity of the pooled estimates. In addition, their analysis focused primarily on skeletal and dental outcomes, with limited evaluation of soft tissue changes. Furthermore, their study did not incorporate structured approaches for assessing the certainty of evidence, such as the GRADE framework, nor did it include comprehensive sensitivity analyzes to explore the robustness of the findings. These methodological differences may partly explain the variation in results and highlight the importance of rigorous study selection and evidence appraisal when interpreting treatment effects in functional orthopedic interventions.

Regarding appliance efficiency, Venkatesan et al. (23) and Cozza et al. (24) reported that Twin Block had higher efficiency among removable appliances (0.46 mm/month), while Herbst led among fixed appliances (0.28 mm/month). Although our analysis did not evaluate efficiency rates, our findings support the orthopedic impact of both appliances. Xinqi et al. (25) noted earlier condylar displacement with Twin Block, absent in Herbst, potentially explaining greater skeletal change in selected cases. Kannan et al. (26) reported that Twin Block significantly increased airway dimensions, especially in the oropharynx and hypopharynx, suggesting added benefits in patients with respiratory concerns.

From a patient experience perspective, Pacha et al. (27) emphasized the lack of data on patient-centered outcomes. D'Antò et al. (28) also highlighted limited methodological quality in previous reviews and the scarcity of strong evidence regarding soft tissue changes. Yirui & Hong (29) concluded that skeletal effects of both appliances are mainly due to ramus growth rather than body elongation, consistent with our lack of significant changes in mandibular body length (e.g., Go-Me).

Timing of treatment relative to the pubertal growth spurt is crucial in functional orthopedics (24). As mandibular peak growth occurs earlier in females (11–12 years) and later in males (12–14 years), asynchrony in treatment initiation could partially explain the variability across studies. Future research should adopt standardized skeletal maturity assessments, such as cervical vertebral maturation, to enhance comparability.

### 4.1 Clinical implications

Although Herbst showed superiority in mentolabial angle and molar correction, these effects were modest. The mentolabial angle affects lower facial aesthetics but changes are often subtle. In contrast, molar correction has greater functional and long-term implications, underscoring its clinical value. Therefore, appliance choice should consider not only skeletal and dental effects, but also patient cooperation, comfort, facial aesthetic needs, and potential airway implications. Our findings do not support universal substitution of one appliance for the other.

### 4.2 Strengths and limitations

This review provides a direct comparison between Herbst and Twin Block, unlike broader reviews including multiple appliances, enhancing its clinical relevance. The inclusion of a quantitative meta-analysis on skeletal, dental, and soft tissue outcomes strengthens its contribution. Rigorous adherence to PRISMA guidelines, assessment of publication bias, and inclusion of recent trials enhance validity. However, limitations include the small number of studies, limiting generalisability; lack of evaluation of patient-reported outcomes and functional variables like airway changes; and heterogeneity in measurement methods and skeletal maturity timing, which may influence findings.

## 5 Conclusion

In conclusion, both the Herbst and Twin Block appliances were efficacious in correcting Class II malocclusion, providing skeletal, dental, and soft tissue improvements under controlled conditions. However, only the mentolabial angle and molar relationship showed statistically significant differences favoring the Herbst appliance. These results were expressed as standarsized mean differences (SMDs), which represent changes in standard deviation units; therefore, their clinical significance should be interpreted cautiously, as statistical significance does not necessarily imply clinical relevance.

Among all outcomes, the molar relationship likely has the greatest clinical impact due to its influence on occlusal function and long-term stability. Nonetheless, no consistent differences were observed in other skeletal or soft tissue variables that would support a universal preference for one appliance over the other.

Therefore, the choice between Herbst and Twin Block should be individualized, considering patient cooperation, comfort, aesthetic and functional needs, and particularly the timing of treatment in relation to the pubertal growth spurt, which varies between sexes and may significantly affect treatment outcomes.

Future research involving larger sample sizes and focused analyzes on skeletal maturation, as well as patient-reported outcomes such as satisfaction and quality of life, will be essential to further refine the selection of the most appropriate functional therapy for Class II malocclusion.

## Data Availability

The original contributions presented in the study are included in the article/Supplementary Material, further inquiries can be directed to the corresponding author.
