# Correction: Herbst and twin block appliances in class II malocclusion management for children: a systematic review and meta-analysis

**DOI:** 10.3389/fdmed.2026.1895861

**Published:** 2026-06-10

**Authors:** Franz Tito Coronel-Zubiate, Joan Manuel Meza-Málaga, Sara Antonieta Luján-Valencia, Consuelo Marroquín-Soto, Fredy Cruzado-Oliva, Rubén Aguirre-Ipenza, Eduardo Luján-Urviola, Carlos Alberto Farje-Gallardo, Ary dos Santos Pinto, Heber Isac Arbildo-Vega

**Affiliations:** 1 Faculty of Health Sciences, Stomatology School, Universidad Nacional Toribio Rodríguez de Mendoza de Amazonas, Chachapoyas, Peru; 2 Faculty of Dentistry, Dentistry School, Universidad Católica de Santa María, Arequipa, Peru; 3 Faculty of Dentistry of Araraquara, Dentistry Science Graduate Program-Orthodontics, São Paulo State University “Júlio de Mesquita Filho” (UNESP), Araraquara, Sao Paulo, Brazil; 4 Department of Diagnostics and Surgery, School of Dentistry, Paulista State University Julio de Mesquita Filho (UNESP), Araraquara, Sao Paulo, Brazil; 5 Department of Dentistry, School of Dentistry, Universidad Científica del Sur, Lima, Peru; 6 Faculty of Stomatology, Stomatology School, Universidad Nacional de Trujillo, Trujillo, Peru; 7 Faculty of Health Sciences, Universidad Continental, Lima, Peru; 8 Faculty of Dentistry, Universidad Andina Néstor Cáceres Velásquez, Juliaca, Peru; 9 Faculty of Dentistry of Araraquara, Department of Morphology and Pediatric Clinic—Orthodontics, São Paulo State University “Júlio de Mesquita Filho” (UNESP), Araraquara, Sao Paulo, Brazil; 10 Faculty of Dentistry, Dentistry School, Universidad San Martín de Porres, Chiclayo, Peru; 11 Faculty of Human Medicine, Human Medicine School, Universidad San Martín de Porres, Chiclayo, Peru; 12 Posgraduate School, Universidad Nacional Toribio Rodríguez de Mendoza de Amazonas, Chachapoyas, Peru

**Keywords:** Class II malocclusion, functional orthodontic appliances, growing patients, Herbst appliance, orthopedic treatment, Twin Block appliance

In the published article, the Funding statement was incorrectly displayed as:

“The author(s) declared that financial support was not received for this work and/or its publication.”

The correct funding statement is:

“This research received no external funding. The Article Processing Charge (APC) was funded by Universidad Nacional Toribio Rodríguez de Mendoza de Amazonas (UNTRM), Peru.”

The original version of this article has been updated.

