# Supplementary material for: Herbst and Twin Block appliances in Class II malocclusion management for children: a systematic review and meta-analysis
Source: Front Dent Med. 2026 May 15;7:1717387. doi: 10.3389/fdmed.2026.1717387 (PMC13219840; doi:10.3389/fdmed.2026.1717387)
Supplement: Supplementary file 1 [file Table1.docx]

Supplementary Table S1. Search strategies for each database

| **Database** |  | **Search Strategy** | **Number of Study** |
| --- | --- | --- | --- |
| **PubMed** | #1 | (((("malocclusion"[MeSH Terms] OR "malocclusion*"[Title/Abstract] OR "tooth crowding*"[Title/Abstract] OR "angle classification"[Title/Abstract] OR "angles classification"[Title/Abstract] OR "malocclusion, angle class ii"[MeSH Terms] OR "angle class ii"[Title/Abstract]) AND "herbst"[Title/Abstract]) OR "herbst appliance*"[Title/Abstract]) AND "twin block"[Title/Abstract]) OR "twin block appliance*"[Title/Abstract] | 266 |
| **Cochrane Library** | #1 | MeSH descriptor: [Malocclusion] explode all trees | 1524 |
|  | #2 | (malocclusion*):ti,ab,kw | 2441 |
|  | #3 | (tooth crowding*):ti,ab,kw | 444 |
|  | #4 | (angle classification):ti,ab,kw | 737 |
|  | #5 | (angles classification):ti,ab,kw | 106 |
|  | #6 | MeSH descriptor: [Malocclusion, Angle Class II] explode all trees | 599 |
|  | #7 | (angle class ii):ti,ab,kw | 789 |
|  | #8 | #1 OR #2 OR #3 OR #4 OR #5 OR #6 OR #7 | 3528 |
|  | #9 | (herbst):ti,ab,kw | 117 |
|  | #10 | (herbst appliance*):ti,ab,kw | 90 |
|  | #11 | #9 OR #10 | 117 |
|  | #12 | (twin block):ti,ab,kw | 244 |
|  | #13 | (twin block appliance*):ti,ab,kw | 177 |
|  | #14 | #12 OR #13 | 244 |
|  | #15 | #8 AND #11 AND #14 | 15 |
| **Web of Science** | #1 | ((((ALL=malocclusion OR (TI=malocclusion* OR AB=malocclusion*) OR (TI="tooth crowding*" OR AB="tooth crowding*") OR (TI="angle classification" OR AB="angle classification") OR (TI="angles classification" OR AB="angles classification") OR ALL="malocclusion, angle class ii" OR (TI="angle class ii" OR AB="angle class ii")) AND (TI=herbst OR AB=herbst)) OR (TI="herbst appliance*" OR AB="herbst appliance*")) AND (TI="twin block" OR AB="twin block")) OR (TI="twin block appliance*" OR AB="twin block appliance*") | 172 |
| **Embase** | #1 | (('malocclusion'/exp OR malocclusion OR malocclusion*:ti,ab OR 'tooth crowding*':ti,ab OR 'angle classification':ti,ab OR 'angles classification':ti,ab OR 'malocclusion, angle class ii'/exp OR 'malocclusion, angle class ii' OR 'angle class ii':ti,ab) AND herbst:ti,ab OR 'herbst appliance*':ti,ab) AND 'twin block':ti,ab OR 'twin block appliance*':ti,ab | 249 |
| **Scopus** | #1 | ( ( ( ( INDEXTERMS ( malocclusion ) OR TITLE-ABS ( malocclusion* ) OR TITLE-ABS ( "tooth crowding*" ) OR TITLE-ABS ( "angle classification" ) OR TITLE-ABS ( "angles classification" ) OR INDEXTERMS ( "malocclusion, angle class ii" ) OR TITLE-ABS ( "angle class ii" ) ) AND TITLE-ABS ( herbst ) ) OR TITLE-ABS ( "herbst appliance*" ) ) AND TITLE-ABS ( "twin block" ) ) OR TITLE-ABS ( "twin block appliance*" ) | 309 |
