# Supplementary material for: Herbst and Twin Block appliances in Class II malocclusion management for children: a systematic review and meta-analysis
Source: Front Dent Med. 2026 May 15;7:1717387. doi: 10.3389/fdmed.2026.1717387 (PMC13219840; doi:10.3389/fdmed.2026.1717387)
Supplement: Supplementary file 2 [file Table2.docx]

Supplementary Table S2. Reason for Exclusion of Full-Text Articles Reviewed for Eligibility

| **Authors** | **Reason for exclusion** |
| --- | --- |
| Siara-Olds et al. [1]  Schaefer et al. [2]  Kannan & Padmanabhan [3]  Deng & Yang [4]  Song et al. [5] | Is not ECA |
| Gu et al. [6] | Assesses the airways |
| Lawton et al. [7] | Population adult patients and students studying apnoea |
| Voudouris & Kuftinec [8] | It does not evaluate the treatment of Class II malocclusion. |
| Batista et al. [9] | Age of the population |

**References**

1. Siara-Olds NJ, Pangrazio-Kulbersh V, Berger J, Bayirli B. Long-term dentoskeletal changes with the Bionator, Herbst, Twin Block, and MARA functional appliances. *Angle Orthodox*. (2010) 80(1):18–29. doi:10.2319/020109-11.1
2. Schaefer AT, McNamara JA Jr, Franchi L, Baccetti T. A cephalometric comparison of treatment with the Twin-block and stainless steel crown Herbst appliances followed by fixed appliance therapy. *Am J Orthod Dentofacial Orthop*. (2004) 126(1):7–15. doi:10.1016/j.ajodo.2003.06.017
3. Kannan A, Padmanabhan S. Comparative evaluation of soft tissue profile changes with Herbst and Twin Block appliances in Class II malocclusion patients: A perception study. *Turk J Orthodox*. (2022) 35(3):173–9. doi:10.5152/TurkJOrthod.2022.21072
4. Deng X, Yang FJ. Orthopedic effects of sagittal-guidance twin-block appliance on facial prognathism. *Int J Clin Exp Med* (2020) 13(1):1–6. https://e-century.us/files/ijcem/13/1/ijcem0096921.pdf
5. Song Y, Yu YL, Shen H, Zhang J. Comparative study of the clinical effects of three different functional appliances on the treatment of skeletal Class II malocclusion. *Hua Xi Kou Qiang Yi Xue Za Zhi.* (2008) 26(4):406–8.
6. Gu M, Savoldi F, Hägg U, McGrath CPJ, Wong RWK, Yang Y. Upper airway changes following functional treatment with the Herbst headgear or Twin Block headgear appliance assessed on lateral cephalograms and magnetic resonance imaging. *Scientific World Journal*. (2019) 2019:1807257. doi:10.1155/2019/1807257
7. Lawton HM, Battagel JM, Kotecha B. A comparison of the Twin Block and Herbst mandibular advancement splints in the treatment of patients with obstructive sleep apnoea: a prospective study. *Eur J Orthodox*. (2005) 27(1):82–90. doi:10.1093/ejo/cjh067
8. Voudouris JC, Kuftinec MM. Improved clinical use of Twin-block and Herbst as a result of radiating viscoelastic tissue forces on the condyle and fossa in treatment and long-term retention: growth relativity. *Am J Orthod Dentofacial Orthop*. (2000) 117(3):247–66. doi:10.1016/S0889-5406(00)70231-9
9. Batista KB, Thiruvenkatachari B, Harrison JE, O’Brien KD. Orthodontic treatment for prominent upper front teeth (Class II malocclusion) in children and adolescents. *Cochrane Database Syst Rev.* (2018) 2018(3):CD003452. doi:10.1002/14651858.CD003452.pub4
