# Supplementary material for: Herbst and Twin Block appliances in Class II malocclusion management for children: a systematic review and meta-analysis
Source: Front Dent Med. 2026 May 15;7:1717387. doi: 10.3389/fdmed.2026.1717387 (PMC13219840; doi:10.3389/fdmed.2026.1717387)
Supplement: Supplementary file 3 [file Table3.docx]

Supplementary Table S3. Methodological and clinical characteristics of the randomized controlled trials included in the systematic review.

| Author(s) | Year | Country | Study Design | Number of patients | Female (F) / Male (M) | Chronological  age in baseline  (in years) | Skeletal age in  baseline (in years) | Follow-up | Observation period | Groups |
| --- | --- | --- | --- | --- | --- | --- | --- | --- | --- | --- |
| Baysal & Uysal [1] | 2011 | Turkey | RCT Parallel | 60 | 11 F /9 M | 12,74 ± 1,43 | 12.20 ± 0.82 | 15,81±5,96 months | Pre-treatment (T0) and post-treatment (T1) | Herbst (HDA) |
|  |  |  |  |  | 10 F /10 M | 13,0 ± 1,32 | 12.26 ± 0.79 | 16,20±7,54 months |  | Twin Block (TB) |
|  |  |  |  |  | 9 F / 11 M | 12,17 ± 1,47 | 11.45 ± 0.95 | 15,58±3,13 months |  | Control Group (CG) |
| Baysal & Uysal [2] | 2013 | Turkey | RCT Parallel | 67 | 11 F /9 M | 12,74 ± 1,43 | 12.20 ± 0.82 | 15,81±5,96 months | T0 (before appliance placement) and T1 (after achieving Class I or super Class I molar relationship) | Herbst (HDA) |
|  |  |  |  |  | 10 F /10 M | 13,0 ± 1,32 | 12.26 ± 0.79 | 16,20±7,54 months |  | Twin Block (TB) |
|  |  |  |  |  | 9 F / 11 M | 12,17 ± 1,47 | 11.45 ± 0.95 | 15,58±3,13 months |  | Control Group (CG) |
| Brandão NMCB. et al. [3] | 2024 | Brazil | RCT Parallel | 30 | 3 F (33.3) /6 M (66,6) | 12,7 ± 1,18 | NR | 12 months | CT imaging: T0 (pre- 12 months treatment) and T1 (after 12 months of treatment) | Herbst (HDA) |
|  |  |  |  |  | 3 F (50) / 3 M (50) | 11.8 ± 1.36 | NR |  |  | Herbst (HSA) |
|  |  |  |  |  | 4 F (40) / 6 M (60) | 12.2 ± 0.80 | NR |  |  | Twin Block (TB) |
| Güler & Malkoc [4] | 2020 | Turkey | RCT Parallel | 60 | 12 F / 8 M | 13.11 ± 2.22 | NR | 9.15 ± 0.93 | Pre-treatment (T0) and post-treatment (T1) | Herbst (HDA) |
|  |  |  |  |  | 12 F /8 M | 12.35 ± 1.01 | NR | 9.55 ± 1.46 |  | Twin Block (TB) |
|  |  |  |  |  | 11 F /9 M | 12.95 ± 0.82 | NR | 9.35 ± 0.93 |  | Monobloque (MB) |
| O’Brien K. et al. [5] | 2003 | United Kingdom | RCT Parallel | 215 | 55 F / 50 M | 12,74 (95% CI: 12,48-12,99) | NR |  | Pre-treatment (T0) and post-treatment (T1) | Herbst (HDA) |
|  |  |  |  |  | 62 F / 48 M | 12,41 (95% CI: 12,17-12,64 | NR |  |  | Twin Block (TB) |
| Pacha M. et al. [6] | 2023 | United Kingdom | RCT Parallel | 80 | 20 F / 20 M | 12.7 ± 1.2 | NR | 3 months | Pre-treatment (T0) and post-treatment (T1) | Hanks Herbst (HDA) |
|  |  |  |  |  | 20 F / 20 M | 12.8 ± 1.3 | NR |  |  | Twin-block (TB) |
| Pacha M. et al. [7] | 2024 | United Kingdom | RCT Parallel | 80 | 20 F / 20 M | 12.7 ± 1.2 | NR | 12 months | Pre-treatment (T0) and post-treatment (T1) | Hanks Herbst (HDA) |
|  |  |  |  |  | 20 F / 20 M | 12.8 ± 1.3 | NR |  |  | Twin-block (TB) |

**References**

1. Baysal A, Uysal T. Soft tissue effects of Twin Block and Herbst appliances in patients with Class II division 1 mandibular retrognathy. *Eur J Orthodox.* (2013) 35(1):71–81. doi:10.1093/ejo/cjq187
2. Baysal A, Uysal T. Dentoskeletal effects of Twin Block and Herbst appliances in patients with Class II division 1 mandibular retrognathy. *Eur J Orthodox*. (2014) 36(2):164–72. doi:10.1093/ejo/cjt013
3. Brandão NMCB, Palomares NB, Lima T, Quintão CCA, Lopes KB, Miguel JAM. Facial soft tissue changes in adolescent patients treated with three different functional appliances: a randomized clinical trial. *Dent Press J Orthod*. (2024) 29(5):e242440. doi: 10.1590/2177-6709.29.5.e242440.oar
4. Güler ÖÇ, Malkoç S. Comparison of facial soft tissue changes after treatment with three different functional appliances. *Am J Orthod Dentofacial Orthop.* (2020)158(4):518–26. doi: 10.1016/j.ajodo.2019.06.020
5. O’Brien K, Wright J, Conboy F, Sanjie Y, Mandall N, Chadwick S, et al. Effectiveness of treatment for Class II malocclusion with the Herbst or Twin-block appliances: a randomized controlled trial. *Am J Orthod Dentofacial Orthop.* (2003)124(2):128–37. doi:10.1016/S0889-5406(03)00345-7
6. Pacha MM, Fleming PS, Pandis N, Shagmani M, Johal A. The use of the Hanks Herbst vs Twin-block in Class II malocclusion: a randomized controlled trial. *Am J Orthod Dentofacial Orthop*. (2023) 164(3):314–24.e1. doi: 10.1016/j.ajodo.2023.06.002
7. Pacha MM, Fleming PS, Shagmani M, Johal A. The skeletal and dental effects of Hanks Herbst versus Twin Block appliances for Class II correction in growing patients: a randomized clinical trial. *Eur J Orthodox*. (2024) 46(1). doi:10.1093/ejo/cjad065
