# Supplementary material for: Herbst and Twin Block appliances in Class II malocclusion management for children: a systematic review and meta-analysis
Source: Front Dent Med. 2026 May 15;7:1717387. doi: 10.3389/fdmed.2026.1717387 (PMC13219840; doi:10.3389/fdmed.2026.1717387)
Supplement: Supplementary file 4 [file Table4.docx]

Supplementary Table S4. Sensitivity Analysis of Selected Variables Under Fixed-Effects and Random-Effects Models

| **Evaluated Variable** | **Fixed Model** | **Random Model** | **Change with Random Model** | **Interpretation** |
| --- | --- | --- | --- | --- |
| Soft tissue convexity (na-prn-pog) | SMD = -0.44 [-0.84, -0.03], p=0.03; Significant (fav. Twin Block) | SMD = -0.41 [-0.91, 0.09], p=0.10; Not significant | Loses significance | Result sensitive to model, finding not robust. |
| VRL – si | SMD = -0.52 [-1.04, 0.00], p=0.05; Borderline significant (fav. Twin Block) | SMD = -0.48 [-1.16, 0.20], p=0.16; Not significant | Loses significance | Result sensitive to model, weak evidence. |
| VRL – pog | SMD = -0.54 [-1.07, -0.02], p=0.04; Significant (fav. Twin Block) | SMD = -0.50 [-1.19, 0.19], p=0.16; Not significant | Loses significance | Result sensitive to model, finding not robust. |

*Comparison of effect sizes and significance levels between fixed-effects and random-effects models for selected outcomes. Values losing statistical significance under the random-effects model indicate sensitivity to heterogeneity and reduced robustness of findings. SMD = Standardized Mean Difference; VRL = Vertical Reference Line.*
