# Supplementary material for: Herbst and Twin Block appliances in Class II malocclusion management for children: a systematic review and meta-analysis
Source: Front Dent Med. 2026 May 15;7:1717387. doi: 10.3389/fdmed.2026.1717387 (PMC13219840; doi:10.3389/fdmed.2026.1717387)
Supplement: Supplementary file 5 [file Table5.docx]

Supplementary Table S5. Reference Norms for Cephalometric and Soft Tissue Parameters

This table summarizes the normal reference values for the cephalometric and soft tissue variables included in the meta-analysis. Values were extracted from the included studies and standardized for comparison.

| Parameter | Normal Value |
| --- | --- |
| Soft tissue convexity (na-prn-pog) | 13°–17° |
| Convexity without nose (na-sn-pog) | 10°–12° |
| H-angle (Holdaway angle) | 10°–12° |
| Nasolabial angle (c-sn-ls) | 102° ± 8° |
| Mentolabial angle (li-sl-pog) | 124° ± 10° |
| VRL – prn | 3–5 mm |
| VRL – sn | 0 mm |
| VRL – ss | 0–2 mm |
| VRL – ls | 2–4 mm |
| E – ls | -4 a -6 mm |
| Basic upper lip thickness | 12–14 mm |
| Upper lip thickness | 13–15 mm |
| Lip strain | 0–2 mm |
| Upper lip length (sn – uls) | 22–24 mm |
| Interlabial gap | 3–4 mm |
| VRL – li | 0–2 mm |
| VRL – si | 0 mm |
| E – li | -2 a -4 mm |
| VRL – pog | -2 a -4 mm |
| Pog – pog | 0 mm |
| si – B | 0–2 mm |
| Lower lip thickness | 14–16 mm |
| Lower lip length (lls – me) | 40–45 mm |
| Molar relationship (is/OLp - Li/OLp) | Forward displacement of the upper incisor relative to the lower incisor indicates Class II malocclusion. |
| Molar relationship (ms/OLp - mi/OLp) | Forward displacement of the upper molar relative to the lower molar indicates Class II malocclusion. |
| Maxillary base (punto A/OLp) | Forward position in Class II. |
| Mandibular base (pg/OLp) | Retruded position in Class II. |
| Skeletal discrepancy (A point to OLp Pg/Olp) | Aumentada en Clase II por discrepancia anteroposterior. |
| Condylar head (co/Olp) | 22 mm |
| Composite mandibular length (pg/OLP+co/OLp) | 120–130 mm |
