# Supplementary material for: Herbst and Twin Block appliances in Class II malocclusion management for children: a systematic review and meta-analysis
Source: Front Dent Med. 2026 May 15;7:1717387. doi: 10.3389/fdmed.2026.1717387 (PMC13219840; doi:10.3389/fdmed.2026.1717387)
Supplement: Supplementary file 6 [file Table6.docx]

Supplementary Table S6. Baseline Cephalometric Dental Measurements Reported in the Included Studies.

| Author(s) | Year | Groups | Number of patients per group | Baseline dental cephalometric measurements | | | | | | | | | | | | | | | |
| --- | --- | --- | --- | --- | --- | --- | --- | --- | --- | --- | --- | --- | --- | --- | --- | --- | --- | --- | --- |
|  |  |  |  | Molar relationship (is/OLp - Li/OLp) | | Molar relationship (ms/OLp - mi/OLp) | | Maxillary base (point A/OLp) | | Mandibular base (pg/OLp) | | Skeletal discrepancy (point A to OLp Pg/Olp) | | Condilar head (co/Olp) | | | Composite mandibular length (pg/OLP + co/OLp) | | |
|  |  |  |  | TF | | TF | | TF | | TF | | TF | | TF | | | TF | | |
|  |  |  |  | Mean | SD | Mean | SD | Mean | SD | Mean | SD | Mean | SD | Mean | SD | Mean | | SD |  |
|  |  |  |  |  |  |  |  |  |  |  |  |  |  |  |  |  | |  |  |
| O’Brien K. et al. | 2003 | Herbst (HDA) | 98 | 3.53 | +3.20 to +3.85 | —1.76 | —2.35 to —1.17 | 73.33 | +72.06 to +74.60 | 76.22 | +74.55 to +77.88 | —2.89 | —3.68 to —1.92 | —13.52 | —14.23 to —12.80 | 62.7 | | +60.83 to +64.56 |  |
|  |  | Twin Block (TB) | 85 | 4.05 | +3.42 to +4.68 | —1.66 | —2.29 to —1.02 | 73.31 | +72.11 to 74.51 | 76.14 | +74.65 to +77.64 | —2.83 | —3.68 to +1.99 | —14.36 | —15.14 to —13.57 | 61.78 | | +60.01 to +63.56 |  |
| Pacha M. et al. | 2024 | Hanks Herbst (HDA) | 40 | 3.6 | 2.2 | (−)5 | 3.2 | 72.8 | 5.3 | 74.1 | 5.5 | (−)1.4 | 3.5 | 13.6 | 3.7 | 87.7 | | 5 |  |
|  |  | Twin-block (TB) | 40 | 5.2 | 3.9 | (−)3.6 | 3.5 | 72.7 | 5.6 | 74.6 | 5.6 | (−)2 | 4.8 | 14.8 | 4 | 89.4 | | 5.7 |  |

TF = Final Time; SD = Standard Deviation; VRL = Vertical Reference Line; CG = Control Group; HDA = Herbst Dental Anchorage; HSA = Herbst Skeletal Anchorage; TB = Twin Block. See Methods for definitions of measurement points.
