# Supplementary material for: Herbst and Twin Block appliances in Class II malocclusion management for children: a systematic review and meta-analysis
Source: Front Dent Med. 2026 May 15;7:1717387. doi: 10.3389/fdmed.2026.1717387 (PMC13219840; doi:10.3389/fdmed.2026.1717387)
Supplement: Supplementary file 7 [file Table7.docx]

Supplementary Table S7. Summary of Meta-Analysis Outcomes for Soft Tissue, Skeletal, and Dental Measurements Between Herbst and Twin Block Appliances

| **Variable / Outcome measured** | **Number of studies** | **Total sample size (H/TB)** | **SMD (IC 95%)** | **Z / p-value** | **I² (%)** | **Heterogeneity** | **Result** |
| --- | --- | --- | --- | --- | --- | --- | --- |
| Soft tissue convexity (na–prn–pog) | 3 | 99 (49 / 50) | -0.41 [-0.91, 0.09] | Z=1.62, p=0.10 | 33 | Low | NS |
| Convexity without nose (na–sn–pog) | 3 | 99 (49 / 50) | -0.53 [-1.20, 0.13] | Z=1.57, p=0.12 | 60 | Moderate | NS |
| H-angle | 3 | 99 (49 / 50) | 0.15 [-0.25, 0.55] | Z=0.74, p=0.46 | 0 | None | NS |
| Nasolabial angle (c–sn–ls) | 3 | 99 (49 / 50) | 0.01 [-0.38, 0.41] | Z=0.07, p=0.95 | 0 | None | NS |
| Mentolabial angle (li–sl–pog) | 3 | 99 (49 / 50) | 0.56 [0.15, 0.96] | Z=2.71, p=0.007 | 0 | None | Significant |
| VRL – prn (Vertical Reference Line to Pronasale) | 2 | 59 (29 / 30) | 0.00 [-0.51, 0.52] | Z=0.02, p=0.99 | 0 | None | NS |
| VRL – sn (Vertical Reference Line to Subnasale) | 2 | 59 (29 / 30) | -0.10 [-0.61, 0.41] | Z=0.40, p=0.69 | 0 | None | NS |
| VRL – ss (Vertical Reference Line to Subspinale) | 2 | 59 (29 / 30) | -0.23 [-0.74, 0.28] | Z=0.88, p=0.38 | 0 | None | NS |
| VRL – ls (Vertical Reference Line to Labrale Superius) | 2 | 59 (29 / 30) | -0.23 [-0.74, 0.28] | Z=0.88, p=0.38 | 0 | None | NS |
| E – ls (E-line to Labrale Superius) | 2 | 59 (29 / 30) | 0.34 [-0.17, 0.86] | Z=1.30, p=0.19 | 0 | None | NS |
| Basic upper lip thickness | 2 | 59 (29 / 30) | 0.11 [-0.41, 0.62] | Z=0.41, p=0.68 | 0 | None | NS |
| Upper lip thickness | 2 | 59 (29 / 30) | -0.27 [-1.20, 0.66] | Z=0.57, p=0.57 | 64 | Moderate | NS |
| Lip strain | 2 | 59 (29 / 30) | 0.18 [-0.33, 0.69] | Z=0.68, p=0.50 | 0 | None | NS |
| Upper lip length (sn–uls) | 2 | 59 (29 / 30) | 0.21 [-0.44, 0.86] | Z=0.64, p=0.52 | 32 | Moderate | NS |
| Interlabial gap | 2 | 59 (29 / 30) | 0.12 [-1.37, 1.60] | Z=0.15, p=0.88 | 85 | High | NS |
| VRL – li (Vertical Reference Line to Labrale Inferius) | 2 | 59 (29 / 30) | -0.30 [-0.82, 0.22] | Z=1.14, p=0.25 | 0 | None | NS |
| VRL – si (Vertical Reference Line to Supramentale Inferius) | 2 | 59 (29 / 30) | -0.48 [-1.16, 0.20] | Z=1.39, p=0.16 | 36 | Moderate | NS |
| E – li (E-line to Labrale Inferius) | 2 | 59 (29 / 30) | 0.29 [-0.42, 0.99] | Z=0.80, p=0.42 | 41 | Moderate | NS |
| VRL – pog (Vertical Reference Line to Pogonion) | 2 | 59 (29 / 30) | -0.50 [-1.19, 0.19] | Z=1.41, p=0.16 | 38 | Moderate | NS |
| Pog – pog (Soft tissue Pogonion to hard tissue Pogonion) | 2 | 59 (29 / 30) | -0.34 [-0.86, 0.17] | Z=1.30, p=0.19 | 0 | None | NS |
| si – B (Supramentale Inferius to point B) | 2 | 59 (29 / 30) | 0.36 [-0.80, 1.53] | Z=0.61, p=0.54 | 77 | High | NS |
| Lower lip thickness | 2 | 59 (29 / 30) | -0.05 [-0.56, 0.46] | Z=0.18, p=0.86 | 0 | None | NS |
| Lower lip length (lls–me) (Labrale Inferius to Menton) | 2 | 59 (29 / 30) | -0.13 [-0.64, 0.38] | Z=0.49, p=0.63 | 0 | None | NS |
| Molar relationship (is/OLp – li/OLp) | 2 | 263 (138 / 125) | -0.31 [-0.56, -0.05] | Z=2.38, p=0.02 | 6 | Low | Significant |
| Deviation towards Class II indicates malocclusion. | 2 | 263 (138 / 125) | -0.19 [-0.55, 0.18] | Z=0.99, p=0.32 | 49 | Moderate | NS |
| Molar relationship (ms/OLp – mi/OLp) | 2 | 263 (138 / 125) | 0.01 [-0.23, 0.25] | Z=0.06, p=0.95 | 0 | None | NS |
| Deviation towards Class II indicates malocclusion. | 2 | 263 (138 / 125) | -0.02 [-0.26, 0.22] | Z=0.16, p=0.87 | 0 | None | NS |
| Maxillary base (point A/OLp) | 2 | 263 (138 / 125) | 0.04 [-0.20, 0.28] | Z=0.31, p=0.75 | 0 | None | NS |
| Forward position in Class II. | 2 | 263 (138 / 125) | -0.01 [-0.54, 0.51] | Z=0.05, p=0.96 | 75 | High | NS |
| Mandibular base (pg/OLp) | 2 | 263 (138 / 125) | -0.07 [-0.47, 0.33] | Z=0.35, p=0.73 | 58 | Moderate | NS |
