# Supplementary material for: Herbst and Twin Block appliances in Class II malocclusion management for children: a systematic review and meta-analysis
Source: Front Dent Med. 2026 May 15;7:1717387. doi: 10.3389/fdmed.2026.1717387 (PMC13219840; doi:10.3389/fdmed.2026.1717387)
Supplement: Supplementary file 8 [file Table8.docx]

Supplementary Table S8. Linear Measurements of Maxillary Soft Tissues Reported in the Included Studies.

| Author(s) | Year | Groups | Number of patients per group | Maxillary soft tissue measurements | | | | | | | | | | | | | | | | | | | | | | |
| --- | --- | --- | --- | --- | --- | --- | --- | --- | --- | --- | --- | --- | --- | --- | --- | --- | --- | --- | --- | --- | --- | --- | --- | --- | --- | --- |
|  |  |  |  | VRL – prn | | VRL – sn | | VRL – ss | | VRL – ls | | E – ls | | | Basic upper lip thickness | | Upper lip thickness | | Lip strain | | Upper lip length: sn – uls | | Interlabial gap | | |  |
|  |  |  |  | TF | | TF | | TF | | TF | | TF | | | TF | | TF | | TF | | TF | | TF | | |  |
|  |  |  |  | Mean | SD | Mean | SD | Mean | SD | Mean | SD | Mean | SD | Mean | | SD | Mean | SD | Mean | SD | Mean | SD | | Mean | SD |  |
|  |  |  |  |  |  |  |  |  |  |  |  |  |  |  | |  |  |  |  |  |  |  | |  |  |  |
| Baysal & Uysal | 2011 | Herbst (HDA) | 20 | 106.82 | 6.88 | 89.65 | 6.14 | 87.07 | 5.14 | 90.70 | 5.71 | -1.45 | 2.50 | 15.10 | | 1.75 | 15.32 | 2.60 | 0.32 | 1.53 | 22.15 | 2.48 | | 1.75 | 2.33 |  |
|  |  | Twin Block (TB) | 20 | 106.2 | 7.56 | 90.4 | 7.64 | 89 | 5.34 | 92.22 | 6.14 | -2.75 | 2.39 | 14.75 | | 1.74 | 14.92 | 2.93 | 0.17 | 2.05 | 22.27 | 3.04 | | 0.22 | 1.00 |  |
|  |  | CG | 20 | 103.62 | 5.45 | 88.1 | 4.56 | 86.4 | 4.54 | 89.82 | 4.17 | 0.67 | 1.57 | 14 | | 1.57 | 13.3 | 2.35 | -0.72 | 1.9 | 21.72 | 2.95 | | 2.47 | 2.96 |  |
| Brandão NMCB. et al. | 2024 | Herbst (HDA) | 9 | 105 | 6.97 | 91.76 | 6.89 | 91.58 | 7.05 | 95.72 | 7.53 | 0.86 | 2.96 | 24.45 | | 3.62 | 13.68 | 1.86 | 10.76 | 3.53 | 25.78 | 2.15 | | 1.06 | 0.94 |  |
|  |  | Herbst (HSA) | 6 | 100.93 | 9.09 | 88.23 | 9.64 | 87.78 | 9.45 | 91.38 | 9.65 | -0.95 | 1.64 | 23.41 | | 3.27 | 14.5 | 1.82 | 8.91 | 3.82 | 24.23 | 2.92 | | 0.71 | 0.84 |  |
|  |  | Twin Block (TB) | 10 | 106.15 | 6.48 | 92.39 | 5.57 | 92.49 | 5.09 | 96.98 | 5.61 | 0.91 | 2.93 | 24.74 | | 2.96 | 15.14 | 1.54 | 9.6 | 2.12 | 23.93 | 3.16 | | 2.60 | 2.83 |  |

TF = Final Time; SD = Standard Deviation; VRL = Vertical Reference Line; CG = Control Group; HDA = Herbst Dental Anchorage; HSA = Herbst Skeletal Anchorage; TB = Twin Block. See Methods for definitions of measurement points.
