# Supplementary material for: Herbst and Twin Block appliances in Class II malocclusion management for children: a systematic review and meta-analysis
Source: Front Dent Med. 2026 May 15;7:1717387. doi: 10.3389/fdmed.2026.1717387 (PMC13219840; doi:10.3389/fdmed.2026.1717387)
Supplement: Supplementary file 9 [file Table9.docx]

Supplementary Table S9. Linear Measurements of Mandibular Soft Tissues Reported in the Included Studies.

| Author(s) | Year | Groups | Number of patients per group | Mandibular soft tissue linear measurements | | | | | | | | | | | | | | | | |
| --- | --- | --- | --- | --- | --- | --- | --- | --- | --- | --- | --- | --- | --- | --- | --- | --- | --- | --- | --- | --- |
|  |  |  |  | VRL – li | | VRL – si | | E – li | | VRL – pog | | Pog – pog | | si – B | | Lower lip thickness | | Lower lip thickness: lls – me | | |
|  |  |  |  | TF | | TF | | TF | | TF | | TF | | TF | | TF | | TF | | |
|  |  |  |  | Mean | SD | Mean | SD | Mean | SD | Mean | SD | Mean | SD | Mean | SD | Mean | SD | Mean | SD |  |
|  |  |  |  |  |  |  |  |  |  |  |  |  |  |  |  |  |  |  |  |  |
| Baysal & Uysal | 2011 | Herbst (HDA) | 20 | 86.05 | 5.76 | 72.85 | 5.45 | 1.32 | 3.12 | 73.80 | 7.22 | 13.85 | 2.55 | 13.42 | 1.61 | 16.50 | 2.42 | 48.50 | 3.69 |  |
|  |  | Twin Block (TB) | 20 | 88.72 | 6.01 | 77.4 | 6.26 | -0.47 | 2.89 | 80.12 | 8.49 | 14.57 | 2.8 | 12.1 | 1.18 | 16.52 | 1.78 | 49.45 | 4.17 |  |
|  |  | CG | 20 | 81.35 | 4.44 | 69.32 | 3.97 | 0.32 | 3.02 | 71.55 | 4.48 | 13.6 | 2.73 | 11.17 | 0.92 | 17.3 | 2.12 | 42.27 | 3.06 |  |
| Brandão NMCB. et al. | 2024 | Herbst (HDA) | 9 | 91.36 | 7.34 | 80.75 | 6.96 | 3.51 | 3.15 | 79.06 | 6.27 | 13.65 | 1.67 | 13.94 | 1.97 | 16.41 | 2.78 | 21.57 | 1.79 |  |
|  |  | Herbst (HSA) | 6 | 88.51 | 10.95 | 78.5 | 9.81 | 2.78 | 3.14 | 79.33 | 8.34 | 14.9 | 1.96 | 13.91 | 1.37 | 16.23 | 2.63 | 19.71 | 2.41 |  |
|  |  | Twin Block (TB) | 10 | 91.38 | 7.06 | 81.18 | 8.2 | 4.11 | 4.27 | 79.72 | 11.67 | 15.72 | 5.04 | 14.71 | 3.14 | 16.81 | 3.35 | 21.17 | 4.93 |  |

TF = Final Time; SD = Standard Deviation; VRL = Vertical Reference Line; CG = Control Group; HDA = Herbst Dental Anchorage; HSA = Herbst Skeletal Anchorage; TB = Twin Block. See Methods for definitions of measurement points.
