# Supplementary material for: Herbst and Twin Block appliances in Class II malocclusion management for children: a systematic review and meta-analysis
Source: Front Dent Med. 2026 May 15;7:1717387. doi: 10.3389/fdmed.2026.1717387 (PMC13219840; doi:10.3389/fdmed.2026.1717387)
Supplement: Supplementary file 10 [file Table10.docx]

Supplementary Table S10. GRADE Evidence Profile for All Outcomes Included in the Meta-Analysis

| **Certainty assessment** | | | | | | | **№ of patients** | | **Effect** | | **Certainty** |
| --- | --- | --- | --- | --- | --- | --- | --- | --- | --- | --- | --- |
| **№ of studies** | **Study design** | **Risk of bias** | **Inconsistency** | **Indirect evidence** | **Imprecision** | **Other considerations** | **[Herbst ]** | **[Twin Block ]** | **Relative (95% CI)** | **Absolute (95% CI)** |  |
| **Soft tissue convexity (na-prn-pog)** | | | | | | | | | | | |
| 3 | Randomized trials | Not serious | Not serious | Not serious | Serious | None | 49 | 50 | - | SMD **0.41 SD lower**  (0.91 higher to 0.09 higher) | ⨁⨁⨁◯ Moderate |
| **Convexity sn nose (na-sn-pog)** | | | | | | | | | | | |
| 3 | Randomized trials | Not serious | Serious | Not serious | Serious | None | 49 | 50 | - | SMD **0.53 SD lower**  (1.2 higher to 0.13 higher) | ⨁⨁◯◯ Low |
| **H angle** | | | | | | | | | | | |
| 3 | Randomized trials | Not serious | Not serious | Not serious | Serious | None | 49 | 50 | - | **0.15 higher** (0.25 lower to 0.55 higher) | ⨁⨁⨁◯ Moderate |
| **Nasolabial angle (c-sn-ls)** | | | | | | | | | | | |
| 3 | Randomized trials | Not serious | Not serious | Not serious | Serious | None | 49 | 50 | - | **0.01 higher** (0.38 lower to 0.41 higher) | ⨁⨁⨁◯ Moderate |
| **Mentolabial angle (li-sl-pog)** | | | | | | | | | | | |
| 3 | Randomized trials | Not serious | Not serious | Serious | Serious | None | 49 | 50 | - | **0.56 higher.** (0.15 higher to 0.96 higher) | ⨁⨁⨁◯ Moderate |
| **VRL – prn** | | | | | | | | | | | |
| 2 | Randomized trials | Not serious | Not serious | Not serious | Serious | None | 29 | 30 | - | **0**  (0.51 lower to 0.52 higher) | ⨁⨁⨁◯ Moderate |
| **VRL – sn** | | | | | | | | | | | |
| 2 | Randomized trials | Not serious | Not serious | Not serious | Serious | None | 29 | 30 | - | **0.1 lower**  (0.61 lower to 0.41 higher) | ⨁⨁⨁◯ Moderate |
| **VRL – ss** | | | | | | | | | | | |
| 2 | Randomized trials | Not serious | Not serious | Not serious | Serious | None | 29 | 30 | - | **0.23 lower**  (0.74 lower to 0.28 higher) | ⨁⨁⨁◯ Moderate |
| **VRL – ls** | | | | | | | | | | | |
| 2 | Randomized trials | Not serious | Not serious | Not serious | Serious | None | 29 | 30 | - | **0.23 lower**  (0.74 lower to 0.28 higher) | ⨁⨁⨁◯ Moderate |
| **E – ls** | | | | | | | | | | | |
| 2 | Randomized trials | Not serious | Not serious | Not serious | Serious | None | 29 | 30 | - | **0.34 higher** (0.17 lower to 0.86 higher) | ⨁⨁⨁◯ Moderate |
| **Basic thickness of the upper lip** | | | | | | | | | | | |
| 2 | Randomized trials | Not serious | Not serious | Not serious | Serious | None | 19 | 30 | - | **0.11 higher** (0.41 lower to 0.62 higher) | ⨁⨁⨁◯ Moderate |
| **Thickness of the upper lip** | | | | | | | | | | | |
| 2 | Randomized trials | Not serious | Very serious | Not serious | Serious | None | 29 | 30 | - | **0.27 lower**  (1.2 lower to 0.66 higher) | ⨁◯◯◯ Very low |
| **Lip tension** | | | | | | | | | | | |
| 2 | Randomized trials | Not serious | Not serious | Not serious | Serious | None | 29 | 30 | - | **0.18 higher** (0.33 lower to0.69 higher) | ⨁⨁⨁◯ Moderate |
| **Upper lip length: sn – uls** | | | | | | | | | | | |
| 2 | Randomized trials | Not serious | Not serious | Not serious | Serious | None | 29 | 30 | - | **0.21 higher** (0.44 lower to 0.86 higher) | ⨁⨁⨁◯ Moderate |
| **Interlabial space** | | | | | | | | | | | |
| 2 | Randomized trials | Not serious | Very serious | Not serious | Serious | None | 29 | 30 | - | **0.12 higher** (1.37 lower to 1.6 higher) | ⨁◯◯◯ Very low |
| **VRL – li** | | | | | | | | | | | |
| 2 | Randomized trials | Not serious | Not serious | Not serious | Serious | None | 29 | 30 | - | **0.3 lower**  (0.82 lower 0.22 higher) | ⨁⨁⨁◯ Moderate |
| **VRL – si** | | | | | | | | | | | |
| 2 | Randomized trials | Not serious | Not serious | Not serious | Serious | None | 29 | 30 | - | **0.48 lower**  (1.16 lower to 0.2 higher) | ⨁⨁⨁◯ Moderate |
| **E – li** | | | | | | | | | | | |
| 2 | Randomized trials | Not serious | Serious | Not serious | Serious | None | 29 | 30 | - | **0.29 higher** (0.42 lower to 0.99 higher) | ⨁⨁◯◯ Low |
| **VRL – pog** | | | | | | | | | | | |
| 2 | Randomized trials | Not serious | Not serious | Not serious | Serious | None | 29 | 30 | - | **0.5 lower**  (1.19 lower to 0.19 higher) | ⨁⨁⨁◯ Moderate |
| **Pog – pog** | | | | | | | | | | | |
| 2 | Randomized trials | Not serious | Not serious | Not serious | Serious | None | 29 | 30 | - | **0.34 lower**  (0.86 lower to 0.17 higher) | ⨁⨁⨁◯ Moderate |
| **si – B** | | | | | | | | | | | |
| 2 | Randomized trials | Not serious | Not serious | Not serious | Serious | None | 29 | 30 | - | **0.36 higher** (0.8 lower to 1.53 higher) | ⨁◯◯◯ Very low |
| **Thickness of the lower lip** | | | | | | | | | | | |
| 2 | Randomized trials | Not serious | Not serious | Not serious | Serious | None | 29 | 30 | - | **0.05 lower**  (0.56 lower 0.46 higher) | ⨁⨁⨁◯ Moderate |
| **Length of lower lip: lls – me** | | | | | | | | | | | |
| 2 | Randomized trials | Not serious | Not serious | Not serious | Serious | None | 29 | 30 | - | SMD **0.13 SD lower** (0.64 higher to 0.38 higher) | ⨁⨁⨁◯ Moderate |
| **Molar ratio (is/OLp - Li/OLp)** | | | | | | | | | | | |
| 2 | Randomized trials | Not serious | Not serious | Serious | Serious | None | 138 | 125 | - | SMD **0.31 SD lower**  (0.56 higher 0.05 lower) | ⨁⨁⨁◯ Moderate |
| **Molar ratio (ms/OLp - mi/OLp)** | | | | | | | | | | | |
| 2 | Randomized trials | Not serious | Serious | Not serious | Serious | None | 138 | 125 | - | SMD **0.19 SD lower** (0.55 higher to 0.18 higher) | ⨁⨁◯◯ Low |
| **Maxillary base (point A/OLp)** | | | | | | | | | | | |
| 2 | Randomized trials | Not serious | Not serious | Not serious | Serious | None | 138 | 125 | - | SMD **0.01 SD higher** (0.23 lower to 0.25 higher) | ⨁⨁⨁◯ Moderate |
| **Mandibular base (pg/OLp)** | | | | | | | | | | | |
| 2 | Randomized trials | Not serious | Not serious | Not serious | Serious | None | 138 | 125 | - | SMD **0.02 SD lower**  (0.26 lower 0.22 higher) | ⨁⨁⨁◯ Moderate |
| **Skeletal discrepancy (A point to OLp Pg/Olp)** | | | | | | | | | | | |
| 2 | Randomized trials | Not serious | Not serious | Not serious | Serious | None | 138 | 125 | - | SMD **0.04 SD higher** (0.2 lower to 0.28 higher) | ⨁⨁⨁◯ Moderate |
| **Condylar head (co/Olp)** | | | | | | | | | | | |
| 2 | Randomized trials | Not serious | Very serious | Not serious | Serious | None | 138 | 125 | - | SMD **0.01 SD lower**  (0.54 lower to 0.51 higher) | ⨁◯◯◯ Very low |
| **Composite mandibular length (pg/OLP+co/OLp)** | | | | | | | | | | | |
| 2 | Randomized trials | Not serious | Serious | Not serious | Serious | None | 138 | 125 | - | SMD **0.07 SD lower**  (0.47 lower 0.33 higher) | ⨁⨁◯◯ Low |

CI: Confidence Inverval; SMD: Standardized mean difference
