# Supplementary material for: Herbst and Twin Block appliances in Class II malocclusion management for children: a systematic review and meta-analysis
Source: Front Dent Med. 2026 May 15;7:1717387. doi: 10.3389/fdmed.2026.1717387 (PMC13219840; doi:10.3389/fdmed.2026.1717387)
Supplement: Supplementary file 11 [file Table11.docx]

Supplementary Table S11. Narrative Summary of GRADE Assessment for Key Outcomes

| Outcome | N° studies | Study design | Risk of bias | Inconsistency | Indirectness | Imprecision | Certainty |
| --- | --- | --- | --- | --- | --- | --- | --- |
| Mentolabial angle (li-sl-pog) | 3 | RCT | No serious | No serious | Serious | Serious | ⨁⨁⨁◯ Moderate |
| Molar relationship (is/OLp - li/OLp) | 2 | RCT | No serious | No serious | Serious | Serious | ⨁⨁⨁◯ Moderate |
| H angle | 3 | RCT | No serious | No serious | No serious | Serious | ⨁⨁⨁◯ Moderate |
| Upper lip thickness | 2 | RCT | No serious | Very serious | No serious | Serious | ⨁◯◯◯ Very low |
| Condylar head (co/OLp) | 2 | RCT | No serious | Very serious | No serious | Serious | ⨁◯◯◯ Very low |

**Abbreviations:**

RCT = Randomized Controlled Trial; OLp = Occlusal Line Perpendicular; li = Labrale Inferius; sl = Sublabiale; pog = Pogonion (soft tissue); is = Incisal edge of upper central incisor; co = Condylion; SMD = Standardized Mean Difference; CI = Confidence Interval.
