# Supplementary material for: Herbst and Twin Block appliances in Class II malocclusion management for children: a systematic review and meta-analysis
Source: Front Dent Med. 2026 May 15;7:1717387. doi: 10.3389/fdmed.2026.1717387 (PMC13219840; doi:10.3389/fdmed.2026.1717387)
Supplement: Supplementary file 12 [file Table12.docx]

Supplementary Table S12. Data Handling and Metric Selection for Pooled Continuous Outcomes

| Study (Author, year) | Outcome | Source table | Imaging modality | Metric pooled | SD source | Notes |
| --- | --- | --- | --- | --- | --- | --- |
| O’Brien et al., 2003 | Molar relationship (is/OLp - Li/OLp) | S6 | Lateral cephalometry | Final values (TF) | Derived from CI | Dental anchorage Herbst |
| O’Brien et al., 2003 | Mandibular base (pg/OLp) | S6 | Lateral cephalometry | Final values (TF) | Derived from CI | - |
| Pacha et al., 2024 | Composite mandibular length | S6 | Lateral cephalometry | Final values (TF) | Reported | - |
| Baysal & Uysal, 2013 | Mentolabial angle (li-sl-pog) | S8 | Lateral cephalometry | Final values (TF) | Reported | - |
| Güler & Malkoc, 2020 | H angle | S8 | Lateral cephalometry | Final values (TF) | Derived from CI | - |
| Brandao et al., 2024 | Mentolabial angle (li-sl-pog) | S8 | CBCT | Final values (TF) | Reported | Excluded in CBCT sensitivity |
| Pacha et al., 2023 | Lower lip thickness | S9 | Lateral cephalometry | Final values (TF) | Derived from SE | _ |

TF = post-treatment (final time). SD = standard deviation. SDs were extracted directly from the original reports when available; when not reported, SDs were derived from confidence intervals (CI) or standard errors (SE) using Cochrane-recommended methods. Change-from-baseline values ​​were not pooled, and no baseline–final correlation assumptions were applied. CBCT = cone-beam computed tomography.
