# Supplementary material for: Herbst and Twin Block appliances in Class II malocclusion management for children: a systematic review and meta-analysis
Source: Front Dent Med. 2026 May 15;7:1717387. doi: 10.3389/fdmed.2026.1717387 (PMC13219840; doi:10.3389/fdmed.2026.1717387)
Supplement: Supplementary file 13 [file Table13.docx]

Supplementary Table S13. Risk of Bias Assessment of Included Randomized Controlled Trials.

| **Sudy** | **D1:**  **Randomization process** | **D2:**  **Deviations from intended interventions** | **D3:**  **Missing outcome data** | **D4:**  **Measurement of the outcome** | **D5:**  **Selection of the reported result** | **Overall risk of bias*** |
| --- | --- | --- | --- | --- | --- | --- |
| Baysal & Uysal (2013) | Low risk | Low risk | High risk | Low risk | High risk | High risk |
| Baysal & Uysal (2014) | Low risk | Low risk | High risk | Low risk | High risk | High risk |
| Brandao et al. (2024) | Low risk | Low risk | Low risk | Low risk | Low risk | Low risk |
| Guler & Malkov (2020) | Low risk | Low risk | Low risk | Low risk | Low risk | Low risk |
| O´Brien et al. (2003) | Low risk | Low risk | Low risk | Low risk | Low risk | Low risk |
| Pacha et al. (2024) | Low risk | Low risk | Low risk | Low risk | Low risk | Low risk |
| Pacha et al. (2023) | Low risk | Some concerns | Low risk | Low risk | Low risk | Some concerns |

* Overall risk-of-bias judgment followed RoB 2.0 guidance: any domain rated High risk → overall High risk; otherwise, any domain rated Some concerns → overall Some concerns; only when all domains are Low risk → overall Low risk.
