# Supplementary material for: Herbst and Twin Block appliances in Class II malocclusion management for children: a systematic review and meta-analysis
Source: Front Dent Med. 2026 May 15;7:1717387. doi: 10.3389/fdmed.2026.1717387 (PMC13219840; doi:10.3389/fdmed.2026.1717387)
Supplement: Supplementary file 14 [file Datasheet1.docx]

Supplementary Figure S1. Forest Plots of All Outcomes

This supplementary file presents all forest plots generated in the meta-analysis comparing Herbst and Twin Block appliances. The included outcomes are grouped into soft tissue, skeletal, and dental domains. Soft tissue measurements encompass soft tissue convexity (na–prn–pog), convexity without nose (na–sn–pog), H angle, nasolabial angle (c–sn–ls), mentolabial angle (li–sl–pog), basic and overall upper lip thickness, lip strain, upper and lower lip length (sn–uls and lls–me), interlabial gap, and vertical reference line distances (VRL) to various landmarks (prn, sn, ss, ls, li, si, pog), as well as E-line distances (E–ls and E–li). Skeletal and dental outcomes include the molar relationship (is/OLp – li/OLp and ms/OLp – mi/OLp), maxillary base (point A/OLp), mandibular base (pg/OLp), skeletal discrepancy (point A to OLp and Pg to OLp), condylar head position (co/OLp), and composite mandibular length (pg/OLp + co/OLp). Figures are presented in the same sequence as in the Results section and Supplementary Tables. All abbreviations are explained in the main manuscript or in Supplementary

**Soft tissue convexity (na-prn-pog)**

**
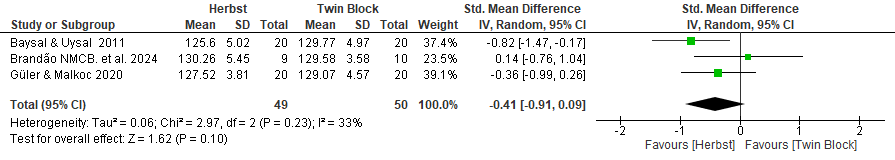
**

**Convexity sn nose (na-sn-pog)**

**
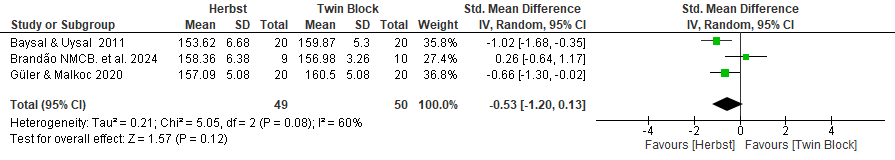
**

**H angle**

**
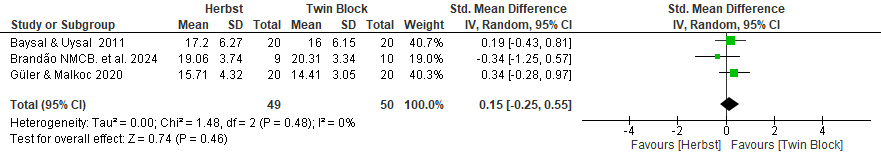
**

**Nasolabial angle (c-sn-ls)**

**
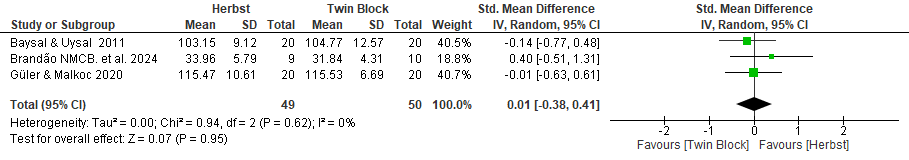
**

**Mentolabial angle (li-sl-pog)**

**
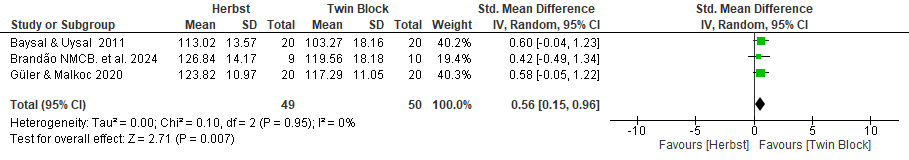
**

**VRL – prn**

**
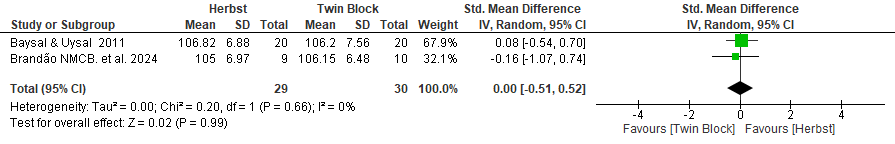
**

**VRL – sn**

**
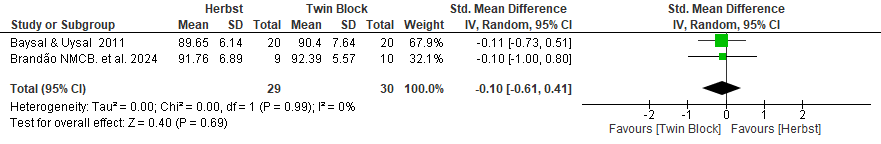
**

**VRL – ss**

**
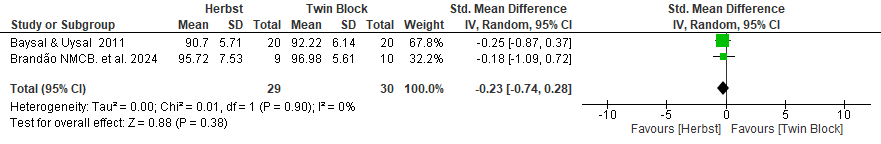
**

**VRL – ls**

**
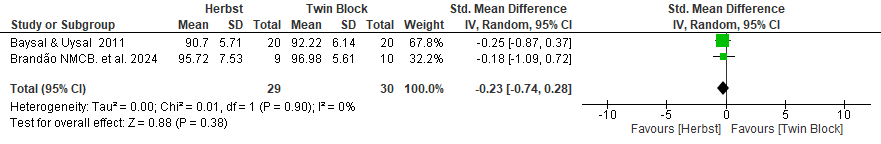
**

**E – ls**

**
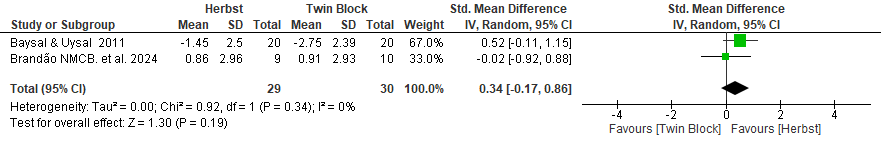
**

**Basic thickness of the upper lip**

**
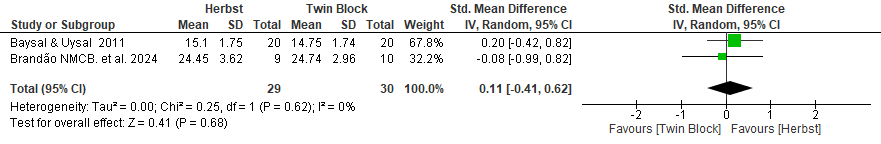
**

**Thickness of the upper lip**

**
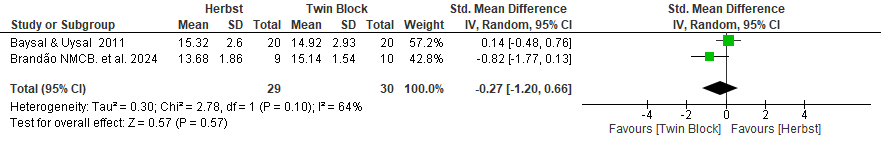
**

**Lip tension**

**
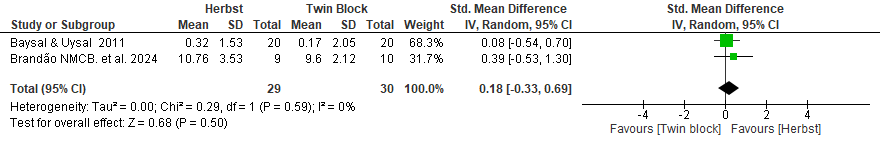
**

**Upper lip length: sn – uls**

**
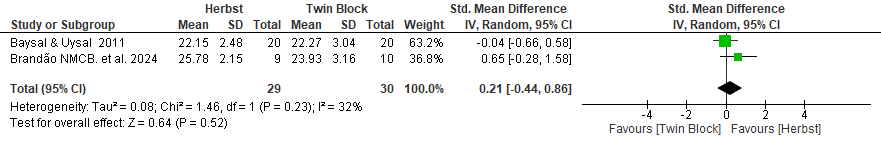
**

**Interlabial space**

**
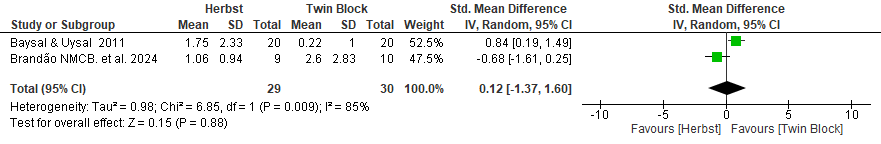
**

**VRL – li**

**
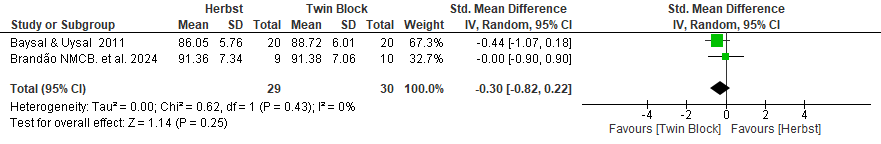
**

**VRL – si**

**
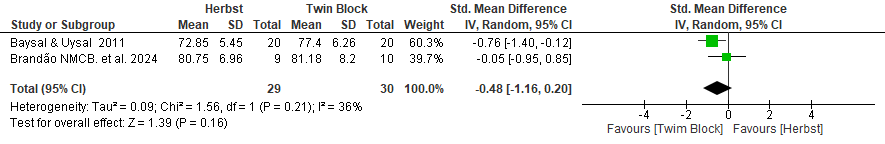
**

**E – li**

**
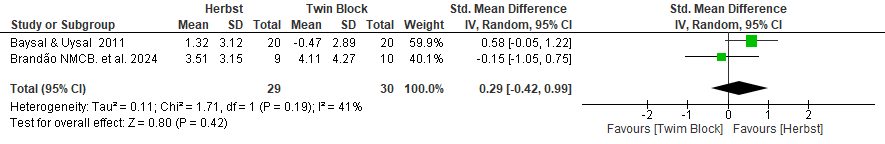
**

**VRL – pog**

**
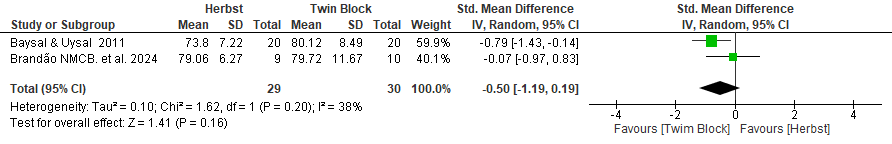
**

**Pog – pog**

**
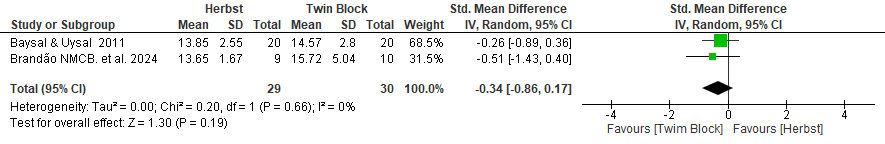
**

**si – B**

**
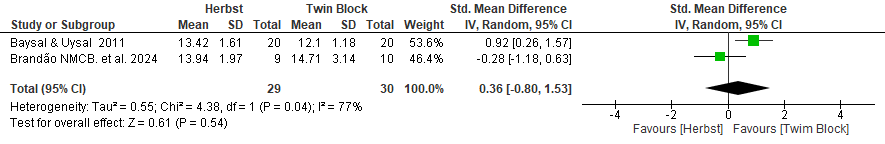
**

**Thickness of the lower lip**

**
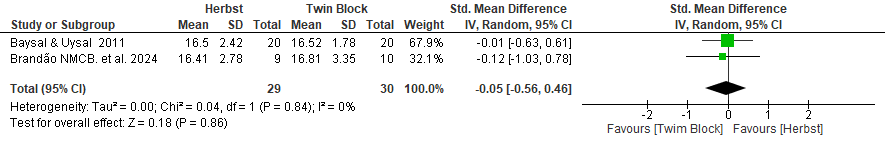
**

**Length of lower lip: lls – me**

**
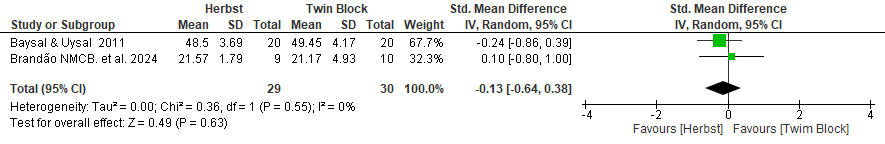
**

**Molar ratio (is/OLp - Li/OLp)**

**
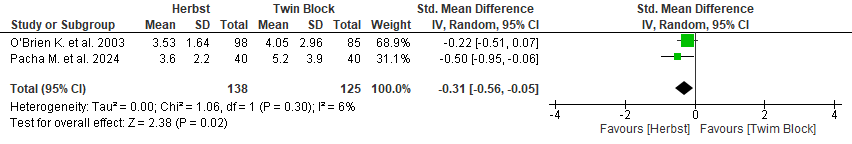
**

**Molar ratio (ms/OLp - mi/OLp)**

**
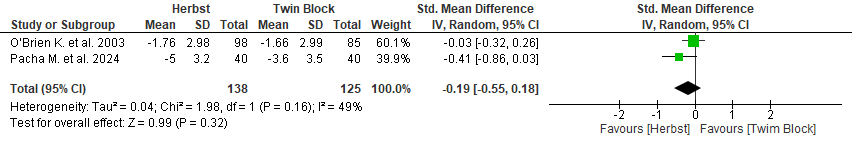
**

**Maxillary base (point A/OLp)**

**
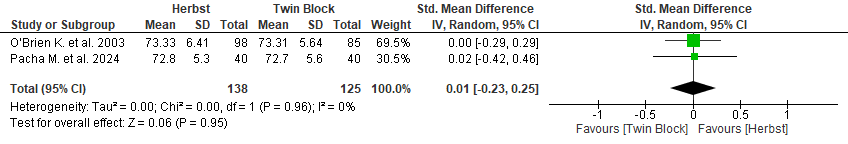
**

**Mandibular base (pg/OLp)**

**
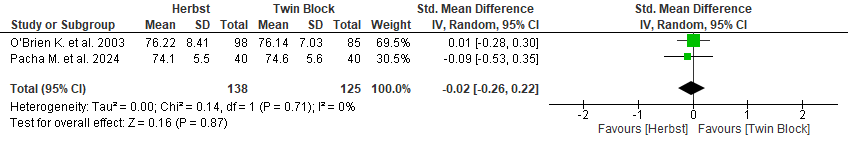
**

**Skeletal discrepancy (A point to OLp Pg/Olp)**

**
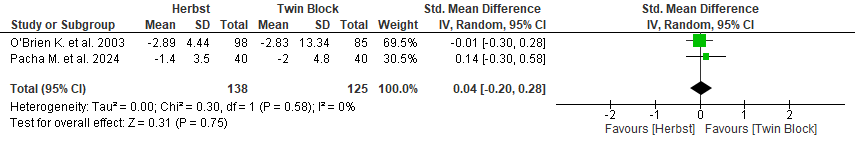
**

**Condylar head (co/Olp)**

**
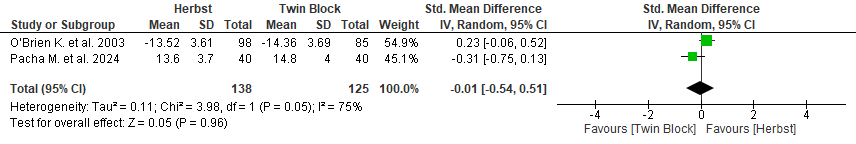
**

**Composite mandibular length (pg/OLP+co/OLp)**

**
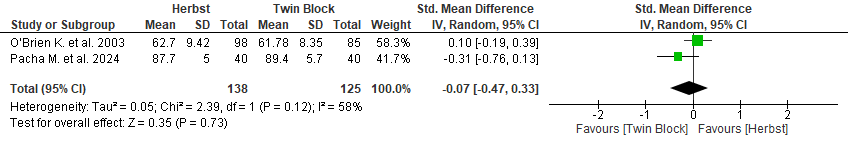
**
